# Inherent tissue homeostasis of the juvenile metaphysis provides a foundation for osteosarcoma development

**DOI:** 10.1038/s41467-026-74929-6

**Published:** 2026-06-26

**Authors:** Masato Saito, Fumie Nakasuka, Nao Sankoda, Yihan Wang, Jumpei Taguchi, Sho Ohta, Yosuke Yamada, Manabu Ozawa, Satoko Sakurai, Atsushi Kondo, Tetsuo Ushiku, Robert Nakayama, Masaya Nakamura, Hiroshi Takayanagi, Atsushi Shibata, Takuya Yamamoto, Yasuhiro Yamada

**Affiliations:** 1 Department of Molecular Pathology, Graduate School of Medicine and Faculty of Medicine, The University of Tokyo, Tokyo, Japan; 2 Department of Orthopedic Surgery, Keio University, School of Medicine, Tokyo, Japan; 3 Core Laboratory for Developing Advanced Animal Models, Center for Experimental Medicine and Systems Biology, Institute of Medical Science, The University of Tokyo, Tokyo, Japan; 4 Department of Life Science Frontiers, Center for iPS Cell Research and Application (CiRA), Kyoto University, Kyoto, Japan; 5 Institute for the Advanced Study of Human Biology (WPI-ASHBi), Kyoto University, Kyoto, Japan; 6 Medical-risk Avoidance Based on iPS Cells Team, RIKEN Center for Advanced Intelligence Project (AIP), Kyoto, Japan; 7 Department of Pathology, Graduate School of Medicine, The University of Tokyo, Tokyo, Japan; 8 Department of Immunology, Graduate School of Medicine and Faculty of Medicine, The University of Tokyo, Tokyo, Japan; 9 Department of Molecular Oncological Pharmacy, Faculty of Pharmacy, Keio University, Tokyo, Japan

**Keywords:** Bone cancer, Stem-cell differentiation

## Abstract

Osteosarcomas preferentially arise in the metaphysis of juvenile long bones, near the growth plate, unlike most cancers, whose incidence increases with age. Here, we show that p21, a negative regulator of the cell cycle, is induced in proliferating juvenile metaphyseal osteoblasts in response to DNA replication-associated damage. Single-cell RNA sequencing defines a differentiation hierarchy from multipotent progenitors to mature osteoblasts and identifies immature osteoblasts enriched for proliferation and replication stress responses. p21-positive metaphyseal osteoblasts associate with growth plate Indian hedgehog expression and decline after growth plate maturation or Hedgehog inhibition. *c-Myc* induction selectively promotes juvenile osteoblast proliferation despite p53 activation, but this proliferative response remains Hedgehog-dependent and ceases after growth plate maturation. By contrast, p53 inactivation enables sustained Hedgehog-independent proliferation of *c-Myc*-induced osteoblasts and lung metastasis. These findings reveal juvenile metaphyseal tissue homeostasis as a potential basis for the age of onset, anatomical specificity, and mutational profile of human osteosarcomas.

## Abstract (web summary)

Osteosarcomas often arise from juvenile metaphysis. Here, the authors show that replication stress-associated p21 expression marks proliferating juvenile osteoblasts within a Hedgehog-dependent niche, while c-Myc activation and p53 loss cooperate to drive Hedgehog-independent osteosarcoma formation.

## Introduction

The incidences of most cancers increase with age, presumably due to the stepwise accumulation of genetic aberrations that are required for cancer development^1,2^. Conversely, pediatric cancers, such as neuroblastomas, arise in early childhood, when residual progenitor cells during development transform into cancer cells based on their intrinsic immature proliferative features^3^. Osteosarcoma is a high-grade bone tumor that predominantly affects the metaphysis of long bones^4,5^. Notably, osteosarcomas often arise in juvenile individuals during the elongation of long bones. However, the underlying mechanisms governing the temporal window of onset and preferential localization of osteosarcoma development remain unclear.

Genetic aberrations in osteosarcomas are characterized by frequent mutations in the *TP53* gene and catastrophic genomic reorganization, including amplifications of oncogenes such as c-*MYC*^6–10^. Previous studies have established various mouse models for osteosarcomas by introducing loss-of-function mutations of *Trp53*^11^. Introduction of *Trp53* mutation in diverse bone lineage cells, ranging from undifferentiated multipotent cells to mature osteoblasts at various differentiation stages, results in osteosarcoma formation^12–16^. A higher incidence of osteosarcoma development has been reported when *Trp53* is disrupted in cells expressing *Osterix* (*Osx*), a marker of immature osteoblasts^12,15,17^. This suggests that *Osx*+ osteoblasts serve as the cells of origin for osteosarcoma development. However, even in these mouse models, the latency of osteosarcoma development often exceeds 1 year^12–15^. Moreover, despite the abundance of *Osx*+ osteoblasts in juvenile bones, osteosarcomas usually develop as a solitary tumor^12^, indicating that other genetic and/or epigenetic events in addition to *Trp53* mutation are required for osteosarcoma development from osteoblasts. Therefore, the sufficient conditions for osteosarcoma development remain to be determined.

In the present study, we established a *p21* reporter mouse model in which cells undergoing DNA damage can be visualized in vivo. Unexpectedly, we found that p21 is expressed in proliferating metaphyseal osteoblasts in juvenile bones as a consequence of DNA damage response (DDR). Single-cell RNA sequencing (scRNA-seq) analysis of the metaphysis at 3 weeks of age uncovered hierarchical differentiation and distinctive proliferative activity accompanied by DDR in juvenile osteoblast lineage cells, which was associated with DNA replication elicited by the unique tissue environment. By leveraging the nature of tissue homeostasis in the juvenile metaphysis, we provide a robust system to study osteosarcoma initiation and unveil a critical role of DDR in the suppression of osteosarcoma.

## Results

### Replication-associated DDR induces *p21* in osteoblasts

**Fig. 1:**
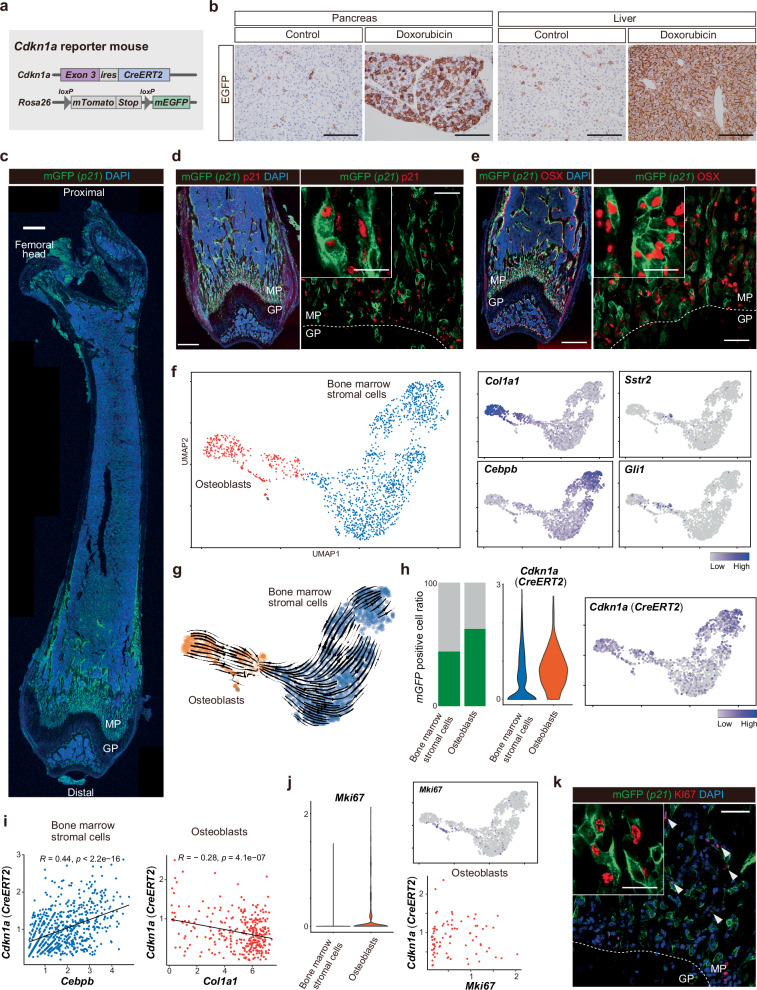
Proliferating juvenile metaphyseal osteoblasts express *p21.* **a** Schematic of the genetic construct used to visualize *p21* expression in vivo. **b** *p21* expression following induction of DNA damage in vivo. **c** mGFP (*p21*) expression in the juvenile femur. MP, metaphysis; GP, growth plate. **d** Metaphyseal cells in juvenile *p21* reporter mice express mGFP and p21 protein. **e** *p21*-mGFP+ metaphyseal cells co-express OSX, an osteoblast marker. **f** Unsupervised clustering of osteoblasts and bone marrow stromal cells from scRNA-seq data. *p21* reporter mice received a single intraperitoneal injection of tamoxifen (1 mg) 48 h before analysis. Right: Expression of canonical marker genes for metaphyseal multipotent progenitors, osteoblasts, and bone marrow stromal cells. **g** RNA velocity analysis of metaphyseal cells. The *Gli1*- or *Sstr2*-expressing cluster shows lineage trajectories toward both osteoblasts and bone marrow stromal cells, consistent with previous lineage-tracing studies identifying these cells as multipotent mesenchymal progenitors. **h** Left: Ratio of *p21*-mGFP+ cells. Middle: *p21* (*CreERT2*) expression levels. Right: UMAP visualization of *p21* (*CreERT2*) expression. **i** Single-cell analysis showing positive correlation between *p21* and *Cebpb* expression in bone marrow stromal cells and negative correlation between *p21* and *Col1a1* expression in osteoblasts. Pearson’s correlation coefficients and *P* values were calculated using the cor.test function in R (version 4.3.2). All tests were two-sided with the null hypothesis of no correlation (*ρ* = 0). **j** Expression of *Mki67* in metaphyseal cells. *p21*-expressing osteoblasts also express *Mki67*. **k** A subset of *p21*+ metaphyseal cells is positive for KI67, a marker of proliferating cells. Scale bars: **b**, 200 μm; **c**–**e**, left, 500 μm, right, 50 μm, inset, 20 μm; **k,** 50 μm; inset, 20 μm. The doxorubicin-treated sample shown in **b** was obtained from a single mouse in one experiment, whereas the control samples were obtained from at least three mice. The experiments shown in **c**–**e** and **k** were repeated independently using at least three mice with similar results.

The cyclin-dependent kinase inhibitor p21 is activated by p53 in response to DNA damage^18–20^. To investigate *p21* expression in vivo, we first established a *p21* reporter mouse model in which *CreERT2* is introduced at the 3’UTR of *Cdkn1a*, together with an *mTmG* reporter allele at a *Rosa26* locus that expresses membrane-targeted GFP as a reporter (Fig. 1a, and Supplementary Fig. 1a). In mice at 6 weeks of age, only a small number of cells expressed mGFP in various organs after tamoxifen treatment (oral gavage, 12.5 mg/kg; Fig. 1b, and Supplementary Fig. 1b), indicating that cells expressing high levels of *p21* are rarely observed under physiological conditions in adult mice. However, 1 week after treatment with doxorubicin, a DNA damage-inducing reagent, the number of mGFP+ cells was substantially increased in many organs, including the liver and pancreas (Fig. 1b, and Supplementary Fig. 1c). Immunohistochemistry confirmed the expression of p21 protein as well as γH2AX in these organs (Supplementary Fig. 1d), indicating that the reporter mouse model successfully visualizes sustained *p21* expression upon DNA damage in vivo.

Unexpectedly, we observed frequent mGFP signals at the metaphysis of long bones in normal juvenile mice (3–4 weeks of age) after tamoxifen treatment (i.p. injection, 100 mg/kg; Fig. 1c, Supplementary Fig. 1e, 2a). mGFP+ cells were also observed at the surface of cortical bone and in a small subset of growth plate chondrocytes in the prehypertrophic zone (Fig. 1c, Supplementary Fig. 2b). Immunofluorescence analysis confirmed the expression of p21 protein in mGFP+ cells (Fig. 1d, Supplementary Fig. 2c). Notably, mGFP+ cells frequently expressed the osteoblast marker OSX (47.4 ± 7.2 %; Fig. 1e) but showed minimal overlap with endomucin (EMCN) or integrin β3 (ITGB3), markers of endothelial and osteoclast cells, respectively (Supplementary Fig. 2d, 2e), demonstrating that mGFP+ cells contain osteoblasts in the juvenile metaphysis. To further validate *p21* expression in osteoblasts, we generated an *Osx* reporter mouse model in which *CreERT2* is inserted after the first ATG of the *Osx* gene and has a *mTmG* reporter allele (Supplementary Fig. 3a). mGFP+ metaphyseal cells in *Osx* reporter mice displayed frequent expression of p21 protein (34.4 ± 5.9 %; Supplementary Fig. 3b–3e), confirming that metaphyseal osteoblasts express *p21*.

To characterize *p21* expression at the single-cell resolution, we next performed single cell RNA-sequencing (scRNA-seq) analysis. Given that *p21*-mGFP+ metaphyseal cells often express OSX, we particularly focused on osteoblasts and bone marrow stromal cells (BMSCs), two major cell types in the metaphysis. These cell populations were isolated from *p21* reporter mice (3 weeks of age) by MACS and FACS (Supplementary Fig. 3f). In a subsequent scRNA-seq analysis, osteoblasts and BMSCs were identified by unsupervised clustering and defined by expression of canonical marker genes (Fig. 1f, and Supplementary Fig. 3g, 3h). A previous study demonstrated that *Gli1*-high metaphyseal cells residing immediately adjacent to the growth plate have multipotent differentiation ability and serve as precursors of both osteoblasts and BMSCs^21^. We successfully identified a *Gli1*-high subpopulation in the osteoblast cluster, which also expressed marker genes previously assigned to multipotent progenitor cells, including *Sstr2*^22^ (Fig. 1f). RNA velocity analysis revealed the transition of *Gli1*/*Sstr2*-high cluster cells into both osteoblasts and BMSCs (Fig. 1g) in line with previous lineage-tracing analyses. *p21*-*mGFP*+ cells were present in both osteoblast and BMSC lineages, but were enriched in osteoblasts, where *p21* expression was markedly higher than in BMSCs (Fig. 1h). BMSCs upregulated *p21* upon differentiation (Fig. 1i), consistent with previous studies linking *p21* expression to differentiation^20^. In sharp contrast, immature osteoblasts expressed higher levels of *p21* than mature osteoblasts (Fig. 1i). *Mki67*+ proliferating cells were preferentially found within osteoblast lineage cells (Fig. 1j), and strikingly, *Mki67*+ osteoblasts often co-expressed *p21* despite its role as a cyclin-dependent kinase inhibitor (Fig. 1j). Immunofluorescence confirmed that a subset of *p21*-mGFP + cells was positive for KI67 (Fig. 1k).

**Fig. 2:**
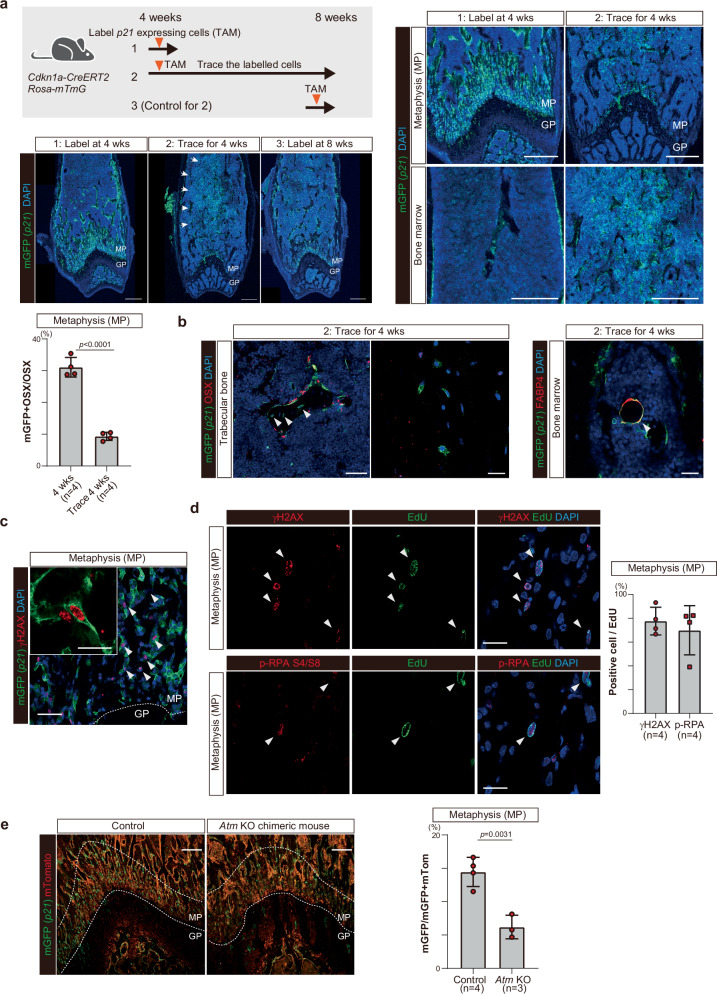
Lineage tracing and replication stress-associated DDR activation in juvenile *p21*+ metaphyseal cells. **a** Upper left: Lineage tracing of *p21*-mGFP+ cells. *p21*-expressing cells were labeled at 4 weeks of age by tamoxifen treatment [1] and traced for 4 weeks [2]. As a control, 8-week-old reporter mice were treated with tamoxifen at 8 weeks of age [3]. Middle left and right: After tracing, mGFP+ cells are reduced in the metaphysis but increased in the bone marrow (arrows). Lower left: Quantification of mGFP+ cells among OSX+ cells. **b** After tracing, a subset of mGFP+ cells gave rise to osteocytes (arrowheads, left) and adipocytes (arrowhead, right). **c** γH2AX expression is detected in a subset of *p21*+ metaphyseal cells. **d** Left: γH2AX and p-RPA expression in EdU+ nuclei. EdU was administered 3 h before sacrifice. Right: Quantification of γH2AX- and p-RPA-positive EdU+ nuclei containing multiple punctate signals. **e** Left: mGFP expression in *p21* reporter chimeric mice lacking *Atm*. Note the reduced proportion of mGFP+ cells in the metaphyseal region. Right: Quantification of mGFP+ cells among metaphyseal cells. The ratio of mGFP+ cells among reporter+ cells (mGFP+ and mTomato+ cells) is shown. Source data are provided as a Source Data file. Data are shown as mean ± SD. For all relevant panels, n indicates the number of mice analyzed. Representative images are from at least three biologically independent mice. Statistical significance was determined by a two-sided unpaired Student’s t-test. The exact *P* value is indicated in the figure. Scale bars: **a,** 500 μm; **b,** left, 50 μm, middle and right, 20 μm; **c**, 50 μm; inset, 20 μm; **d,** 20 μm; **e**, 200 μm. MP metaphysis; GP growth plate.

To further characterize *p21*-mGFP+ metaphyseal cells, we next conducted lineage-tracing analysis. *p21*-mGFP+ cells were labeled at 4 weeks of age and followed for 4 weeks (Fig. 2a). Over time, the number of labeled cells was markedly reduced in the metaphysis (Fig. 2a), indicating that *p21*-mGFP+ cells lack long-term self-renewal capacity and do not represent mesenchymal stem/progenitor cells. Conversely, the number of labeled cells was increased in the bone marrow (Fig. 2a). Immunofluorescence revealed that these cells give rise, at least in part, to differentiated cells, including osteocytes in trabecular bone and adipocytes in marrow (Fig. 2b). Indeed, 58.7 ± 14.5% of FABP4+ cells in marrow exhibited mGFP signals after the chase period, indicating that *p21*-mGFP+ cells differentiated into adipocytes. Collectively, these findings demonstrate the heterogeneous nature of *p21* expression and show that *p21* is expressed in immature, proliferating osteoblast-lineage cells.

We noticed that a subset of *p21*+ cells expressed γH2AX, a marker of the DDR (10.6 ± 1.6 %; Fig. 2c), which raised the possibility that these cells undergo replication stress. Phosphorylated RPA at Ser4/Ser8 (p-RPA) is a marker associated with replication stress and stalled replication forks^23^. Remarkably, we observed frequent p-RPA and γH2AX positivity in EdU-incorporated S-phase nuclei of metaphyseal cells that also expressed OSX (Fig. 2d, and Supplementary Fig. 3i). These findings indicate that proliferating juvenile metaphyseal osteoblasts exhibit DDR during DNA replication^24^. DNA damage signaling is regulated by protein phosphorylation via three related kinases, ATM, ATR, and DNA-PK, which activate various biological processes, including DNA repair, cell cycle control, and apoptosis partly through p53 activation^25^. To confirm the involvement of the DDR in the induction of *p21*, we generated chimeric mice with knockout (KO) of *Atm* (Supplementary Fig. 4a). *Atm* KO chimeric mice showed significantly reduced numbers of mGFP+ cells (Fig. 2e), demonstrating that *p21* is induced, at least in part, by the DDR. Immunohistochemical analysis of human metaphyseal tissues revealed p21 protein expression in proliferating juvenile osteoblasts (*n* = 5, Supplementary Fig. 4b).

### Unique transcriptional profile of juvenile osteoblasts

**Fig. 3:**
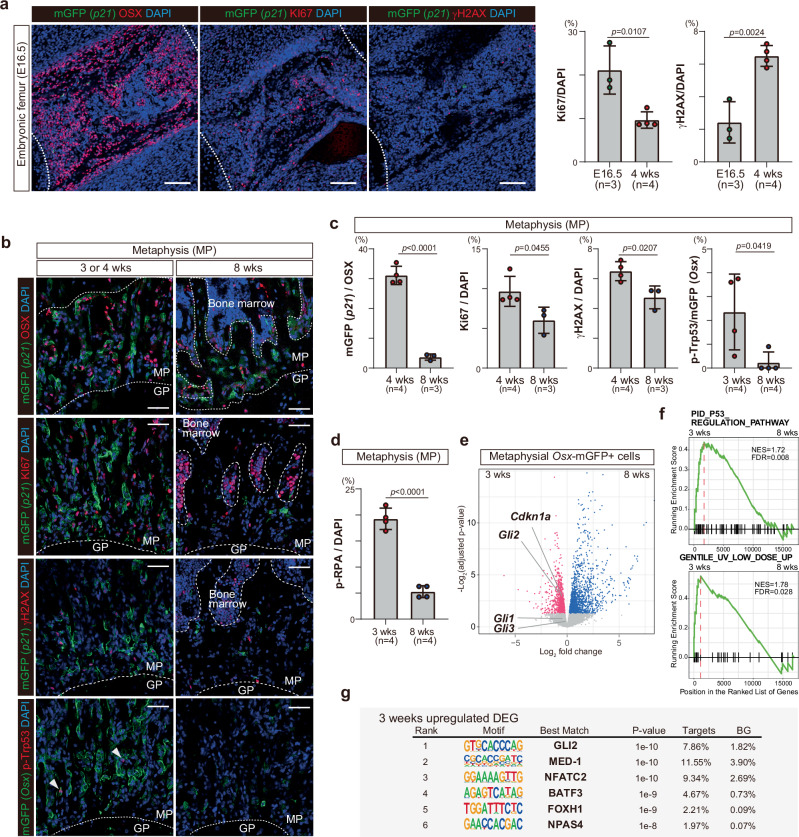
Preferential *p21* expression and a distinct transcriptional profile in juvenile metaphyseal osteoblasts. **a** Expression of mGFP (*p21*) and γH2AX in the embryonic femur (E16.5). Immunofluorescence images (left) and quantification of Ki67- and γH2AX-positive cells (right). **b** Representative immunofluorescence images of the metaphysis in juvenile and adult mice. **c** Quantification of immunofluorescence analysis. Data for Ki67 and γH2AX at 4 weeks of age are identical to those shown in Fig. 3a. **d** Quantification of p-RPA-positive metaphyseal cells. **e** Volcano plot comparing gene expression profiles of *Osx*+ osteoblasts between juvenile (3 weeks old) and adult (8 weeks old) mice. *Osx*+ metaphyseal osteoblasts were collected 2 days after the final tamoxifen treatment administered on 3 consecutive days. Genes with adjusted *p* < 0.05 are highlighted. The x-axis indicates log2 fold change and the y-axis represents significance following DESeq2 testing. **f** GSEA of DNA damage-associated gene sets, indicating enhanced DDR in juvenile osteoblasts. **g** De novo motif analysis of upregulated DEGs in juvenile *Osx*+ osteoblasts (433 genes, adjusted *p* < 0.05). Promoter regions (–400 bp to +100 bp from the transcription start site) were analyzed. The GLI2 motif, a downstream transcription factor of Hedgehog signaling, was significantly enriched. Source data are provided as a Source Data file. For all relevant panels, n indicates the number of mice analyzed. Representative images are from at least three biologically independent mice. Statistical significance was determined by a two-sided unpaired Student’s t-test. The exact *P* value is indicated in the figure. Scale bars: **a**–**c**, 50 μm. MP metaphysis; GP growth plate.

Osteoblasts proliferate actively during skeletal development in embryonic and neonatal mice. To assess the DNA replication-associated DDR in proliferating osteoblasts during development, we examined *p21* and γH2AX expression in osteoblasts of E16.5 femurs from *p21* reporter mice. Despite their active proliferation, both *p21* reporter activity and γH2AX expression were scarcely detected in embryonic osteoblasts (Fig. 3a). Next, we examined *p21* expression in the metaphysis of young adult mice (8 weeks of age; hereafter referred to as adult mice), when longitudinal bone growth is nearing its endpoint. The number of *p21*-mGFP+ metaphyseal cells was substantially reduced in adult mice (Figs. 2a, 3b, c, and Supplementary Fig. 4c), accompanied by decreases in KI67+ and γH2AX+ cells (Fig. 3b, c, Supplementary Fig. 4d). Consistently, phosphorylation of p53 at S18 (equivalent to S15 in human p53), a key target of the DDR, was readily detected in juvenile osteoblasts but was scarcely observed in adult osteoblasts (Fig. 3b, c). Moreover, the frequency of p-RPA-positive cells was markedly reduced in the adult metaphysis (Fig. 3d, Supplementary Fig. 4e). In addition, treatment with the DNA damage-inducing agent doxorubicin further increased the number of p21+ cells specifically in juvenile metaphyseal cells (Supplementary Fig. 4f). Together, these results indicate that DNA replication-associated DDR and *p21* expression preferentially occur in juvenile metaphyseal osteoblasts during physiological bone growth, underscoring the unique properties of juvenile metaphyseal osteoblasts.

To understand the transcriptional profile of juvenile metaphyseal osteoblasts, we next conducted bulk RNA-seq analysis of juvenile (3 weeks of age) and adult (8 weeks of age) osteoblasts. mGFP+ cells in the metaphysis of *Osx* reporter mice were sorted for analysis. We identified differentially expressed genes (DEGs) in juvenile *Osx*+ osteoblasts (433 upregulated DEGs and 814 downregulated DEGs, *p* < 0.05) compared with adult *Osx*+ osteoblasts, which included *p21* (*Cdkn1a*) among upregulated DEGs (Fig. 3e, Supplementary Fig. 4g). In agreement with the immunofluorescence analysis, gene set enrichment analysis (GSEA) suggested that the DDR was increased in juvenile osteoblasts (Fig. 3f). To investigate key transcription factors that characterize the transcriptional profile of juvenile osteoblasts, we next performed motif analysis of DEGs. This identified overrepresentation of the motif for GLI2, a downstream transcription factor in the Hedgehog signaling pathway^26,27^, in the upstream region of upregulated DEGs [−400 bp to +100 bp relative to the transcription start site (TSS)] (Fig. 3g). Furthermore, expression of *Gli2* was significantly elevated in juvenile osteoblasts (Fig. 3e, and Supplementary Fig. 4g, 4h), suggesting that GLI2 establishes unique transcriptional signatures in juvenile metaphyseal osteoblasts. Consistently, *Igf2*, which is reportedly a target of Hedgehog signaling in osteoblasts^28^, was significantly upregulated in juvenile osteoblasts (Supplementary Fig. 4h).

### scRNA-seq reveals osteoblast differentiation hierarchy

We next compared the transcriptional profiles of juvenile and adult metaphyseal cells at single-cell resolution (Supplementary Fig. 5a–c). Guided by the increased expression of *Gli2* and the enrichment of GLI2 motifs among upregulated DEGs in bulk RNA-seq, we first focused on the Hedgehog signaling pathway. Of the three Hedgehog ligands, only *Indian hedgehog* (*Ihh*) was detected, and its expression was restricted to the chondrocyte cluster (Supplementary Fig. 5d). By contrast, expression of *Gli1*, *Gli2*, and *Gli3* was observed primarily in the osteoblast and chondrocyte clusters (Supplementary Fig. 5d).

**Fig. 4:**
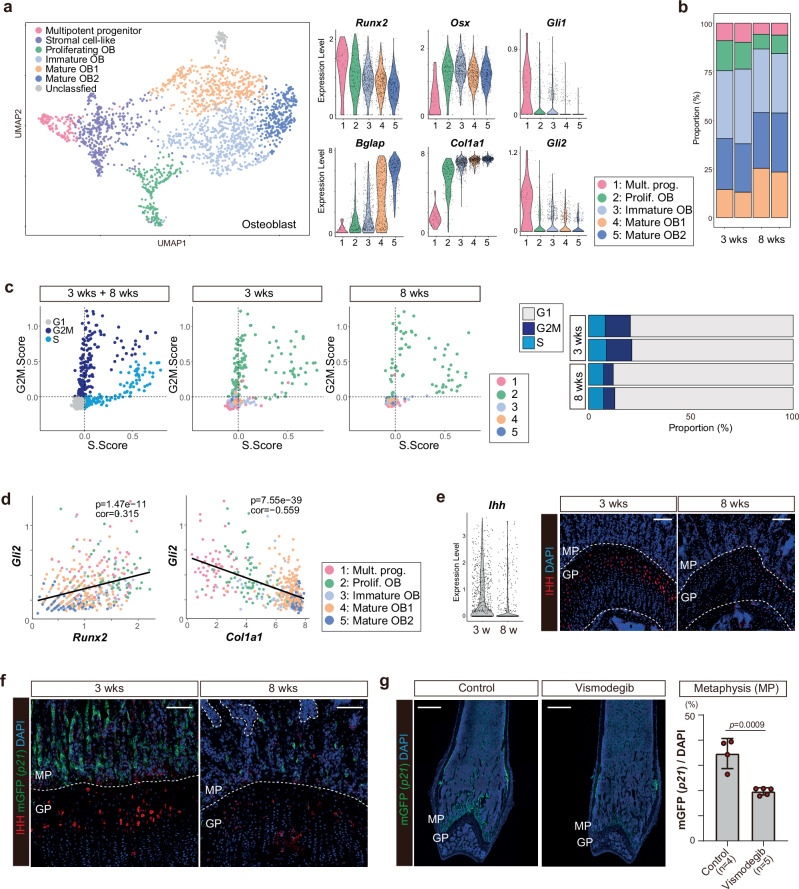
Tissue homeostasis in the juvenile metaphysis revealed by scRNA-seq analysis. **a** Subclustering of osteoblast-lineage cells. Expression of lineage marker genes indicates a differentiation trajectory from multipotent progenitors to mature osteoblasts. **b** Proportions of each osteoblast-lineage cluster in juvenile and adult mice. Clusters of proliferating and immature osteoblasts, as well as multipotent progenitors, are reduced in adults, whereas mature osteoblast clusters are increased. **c** Cell cycle scoring of osteoblast-lineage cells. A high proportion of proliferating osteoblasts are in S and G2/M phases. Right: Distribution of osteoblast-lineage cells across cell cycle phases. Juvenile osteoblasts exhibit an increased proportion of G2/M-phase cells, suggesting prolonged G2/M progression. **d** Single-cell analysis showing a negative correlation between *Gli2* and *Col1a1* expression and positive correlation between *Gli2* and *Runx2* expression. Pearson’s correlation coefficients and *P* values were calculated using the cor.test function in R (version 4.3.2). All tests were two-sided with the null hypothesis of no correlation (*ρ* = 0). **e** Left: *Ihh* expression in juvenile and adult chondrocyte-lineage cells. Right: Immunofluorescence analysis of IHH expression in the metaphysis. In juvenile mice, IHH is predominantly expressed in prehypertrophic chondrocytes of the growth plate, whereas IHH expression is markedly reduced in adult chondrocyte-lineage cells. **f** Immunofluorescence analysis of IHH and mGFP in *p21* reporter mice. The emergence of *p21*+ metaphyseal cells coincides with IHH expression in the growth plate. **g** Vismodegib treatment, a Hedgehog signaling inhibitor, significantly reduces the number of *p21*+ cells. Right: Quantification of *p21*+ cells. Juvenile mice (3 weeks old) were treated with vismodegib (100 mg/kg body weight) five times per week and analyzed on day 7. Statistical significance was determined by a two-sided unpaired Student’s t-test. Source data are provided as a Source Data file. For all relevant panels, *n* indicates the number of mice analyzed. Representative images are from at least three biologically independent mice. The exact *P* value is indicated in the figure. Scale bars: **e,** 100 μm; **f**, 50 μm; **g,** 500 μm. MP metaphysis; GP growth plate.

Subclustering of the osteoblast cluster revealed distinct cell types, including *Gli1*-high multipotent progenitors, BMSC-like cells, and osteoblasts, which were further divided into proliferating, immature, and mature osteoblasts (Fig. 4a, and Supplementary Fig. 6a). Expression of canonical osteoblast markers indicated a differentiation trajectory from multipotent progenitors through proliferating osteoblasts to immature and finally mature osteoblasts (Fig. 4a). Notably, juvenile osteoblasts contained a higher proportion of immature osteoblasts, while adult osteoblasts exhibited a substantially increased proportion of mature osteoblasts (Fig. 4b, and Supplementary Fig. 6b). *Mki67*-expressing cells were more frequently observed in the immature proliferating osteoblast clusters than in the multipotent progenitor population (Supplementary Fig. 6c), suggesting a higher proliferative activity in osteoblasts. Consistently, cell cycle analysis based on phase-specific gene expression revealed that the vast majority of S- and G2/M-phase cells were present among the proliferating osteoblast cluster (Fig. 4c). Of particular note, the proportion of G2/M-phase cells was higher among juvenile osteoblasts than among adult osteoblasts (Fig. 4c), indicating a prolonged G2/M phase in the juvenile population, consistent with p53 activation. Gene Ontology (GO) analysis further indicated that DNA replication was increased and DDR pathways were activated in juvenile proliferating osteoblasts (Supplementary Fig. 6d). Taken together, these findings suggest the presence of DNA replication-associated DDR primarily in juvenile osteoblasts.

Remarkably, *Gli2* expression was highest in *Gli1*-high multipotent progenitors and progressively decreased upon differentiation into osteoblasts (Fig. 4a). Moreover, the *Gli2* expression level was positively correlated with the expression level of *Runx2*, a marker of immature metaphyseal cells, and negatively correlated with the expression level of *Col1a1*, a marker of mature osteoblasts, at the single-cell level (Fig. 4d, and Supplementary Fig. 6e). Considering that RUNX2 expression decreased while COL1A1 expression increased in metaphyseal cells with increasing distance from the growth plate (Supplementary Fig. 6f), our results also indicate that the *Gli1*/*Gli2* expression levels are negatively correlated with the distance from the growth plate.

Subclustering of the chondrocyte cluster revealed that *Ihh* expression is considerably reduced in adult chondrocytes (Fig. 4e, and Supplementary Fig. 7a, 7b). Immunofluorescence analysis confirmed that IHH was expressed at the layer containing prehypertrophic chondrocytes, predominantly in the juvenile growth plate (Fig. 4e, and Supplementary Fig. 7c). Remarkably, elevated IHH expression was associated with the emergence of *p21*+ metaphyseal cells near the growth plate (Fig. 4f). Furthermore, the number of *p21*+ metaphyseal cells was significantly reduced by treatment with vismodegib, an inhibitor of Hedgehog signaling^29^ (Fig. 4g). Together with the progressive reduction of *Gli2* expression in osteoblasts with increasing distance from IHH-expressing cells, our results suggest that Hedgehog signaling in response to chondrocyte-derived IHH at the growth plate maintains the proliferative immature state of metaphyseal osteoblasts.

### *c-Myc* induces transient osteoblast proliferation

We found that juvenile *Osx*+ metaphyseal osteoblasts exhibit distinctive transcriptional features and express *p21* through the DDR. Given that osteosarcomas often arise in the metaphysis of juvenile individuals^30^, we anticipated that juvenile *p21*+ osteoblasts would be the progenitor cells of osteosarcomas. To test this hypothesis, we introduced a dominant-negative mutation of *Trp53* into juvenile *p21*+ cells^31^ (Supplementary Fig. 8a, 8b). After 1 year, a subset of mice developed bone tumors (Supplementary Fig. 8c, 8d). The developed tumors were osteogenic osteosarcomas exhibiting formation of extracellular osteoid (Supplementary Fig. 8e), demonstrating that juvenile *p21*+ cells can transform into osteosarcomas. However, the majority of *Trp53* mutant mice did not develop osteosarcomas even after aging. Moreover, osteosarcomas always arose as a solitary tumor, and the metaphysis exhibited normal histology in most bones, indicating that *Trp53* mutation alone is not sufficient for initiation of *p21*+ cell proliferation.

**Fig. 5:**
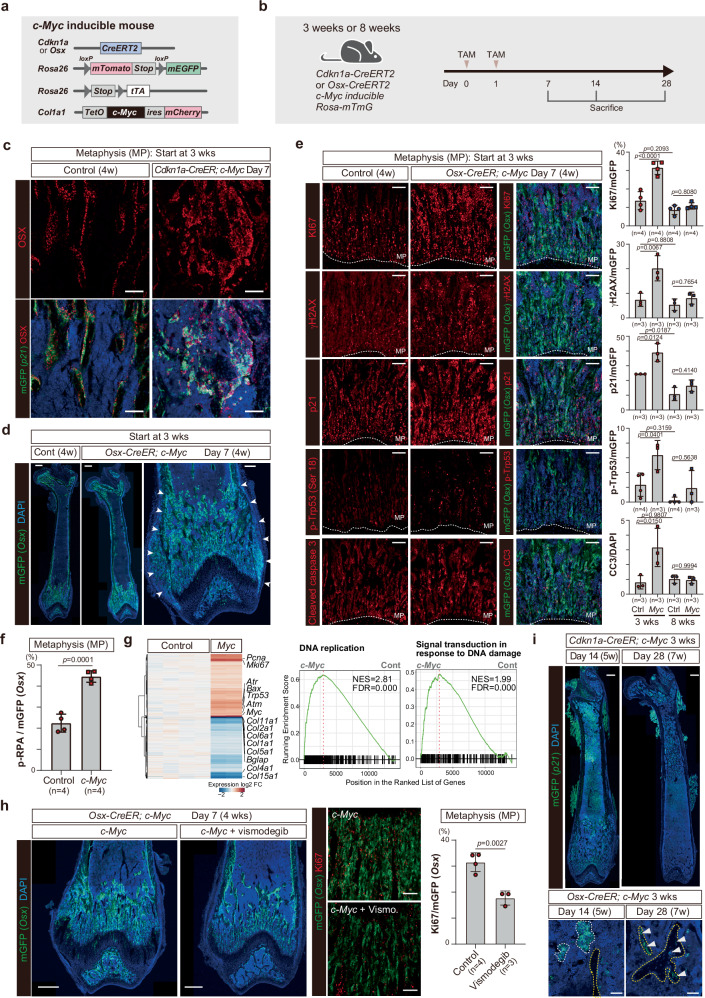
*c-Myc* promotes active proliferation of juvenile osteoblasts but enhances DDR. **a** Schematic of the genetic construct used for spatiotemporal induction of *c-Myc* in osteoblasts. **b** Experimental protocol for ectopic *c-Myc* induction. **c** OSX+ cells are selectively expanded following *c-Myc* induction in the *Cdkn1a-CreERT2* model. **d** Effects of *c-Myc* transduction in the *Osx-CreERT2* model. *c-Myc* induces robust expansion of osteoblasts in juvenile mice with kinetics similar to those observed in the *Cdkn1a-CreERT2* model. Arrows indicate expanded *Osx*+ osteoblasts. **e** Immunofluorescence analysis of *c-Myc*-induced osteoblasts in juvenile mice analyzed 7 days after induction. Right: Quantification of the results. **f** Quantification of p-RPA-positive cells among *Osx*-mGFP+ cells following *c-Myc* induction. Cells containing multiple punctate nuclear p-RPA signals were scored as positive. **g** Left: Heatmap of differentially expressed genes in osteoblasts following *c-Myc* transduction (|log2 fold change | ≥ 1, adjusted *p* < 0.05). *Osx*-mGFP+ metaphyseal cells were collected 7 days after two consecutive tamoxifen treatments. Genes associated with proliferation, DNA damage, and apoptosis are upregulated, whereas differentiation-associated genes are downregulated. Expression values are shown as log2 fold changes relative to controls. Right: GSEA of gene sets associated with DNA replication and DDR. **h** Vismodegib treatment inhibits expansion of *Osx*+ osteoblasts. Left: KI67+ cells are significantly reduced following treatment. Right: Quantification of the results. **i** Upper: Expanded mGFP+ cells are markedly reduced by day 28 in the *Cdkn1a-CreERT2* model. Lower: GFP signal is detected within trabecular bone at day 28, but not day 14, in the *Osx-CreERT2* model. Yellow dashed lines indicate trabecular bone structures. White dashed lines at day 14 outline the area of osteoblast expansion. Source data are provided as a Source Data file. Data are shown as mean ± SD. For all relevant panels, *n* indicates the number of mice analyzed. Representative images are from at least three biologically independent mice. For comparisons between two groups, statistical significance was determined by two-sided unpaired Student’s t-test. For multiple pairwise comparisons among more than two groups, statistical significance was determined by two-sided one-way ANOVA with Tukey’s adjustment for multiple comparisons. Exact *P* values are indicated in the figure. Scale bars: **c**, **e** and **h**, right, 50 μm; **d**, left and middle and **h**, left, 500 μm; **d,** right, 200 μm; **i**, upper, 500 μm **i**, lower, 50 μm.

To delineate the conditions for osteosarcoma initiation, we reviewed genetic aberrations reported in human osteosarcomas. Among them, we focused on *c-Myc* amplification because *c-Myc* induces DNA replication^32^ and is frequently amplified in human osteosarcomas^8^, which could contribute to the initiation of osteoblast proliferation. Remarkably, *c-Myc* induction in juvenile *p21*+ cells resulted in the rapid, selective expansion of OSX+ osteoblasts in the *Cdkn1a-CreERT2* model (Fig. 5a–c, and Supplementary Fig. 9a)^33,34^, indicating that *p21*+ juvenile osteoblasts are permissive for replication signal driven by *c-Myc*. Consistent with this, *c-Myc* induction in *Osx*+ cells phenocopied the rapid proliferation of osteoblasts in the *Osx-CreERT2* model, with kinetics comparable to the *Cdkn1a-CreERT2* model (Fig. 5d, and Supplementary Fig. 9b, c). Active proliferation was initially evident at metaphyseal osteoblasts in the marrow and periosteum, and extended toward the diaphyseal region in 2 weeks (5 weeks of age) (Supplementary Fig. 9b, c). Remarkably, along with active proliferation, expression of DDR-related proteins, such as γH2AX, p21, phospho-p53, and cleaved caspase 3, was augmented in *c-Myc*-induced juvenile osteoblasts (Fig. 5e). Moreover, the increased frequency of p-RPA-positive cells in juvenile osteoblasts was further enhanced after *c-Myc* induction, supporting the notion that *c-Myc* exacerbates replication stress in proliferating juvenile osteoblasts (Fig. 5f, and Supplementary Fig. 9d). Consistently, RNA-seq analyses of *Osx*+ metaphyseal osteoblasts revealed active proliferation and an enhanced DDR in *c-Myc*-induced osteoblasts at Day 7, as shown by increased expression of *Mki67*, *Pcna*, *Atr*, and *Bax* (Fig. 5g). GO analyses and GSEA also suggested that DNA replication and an activated DDR were increased in *c-Myc*-induced osteoblasts (Fig. 5g, and Supplementary Fig. 10a). By contrast, genes associated with osteoblast differentiation, including *Col1a1* and *Bglap*, were downregulated in *c-Myc*-induced osteoblasts (Fig. 5g, and Supplementary Fig. 10a). In sharp contrast, *c-Myc* induction in adult *Osx*+ osteoblasts did not cause active proliferation and induced only modest activation of the DDR (Fig. 5e, and Supplementary Fig. 10b, c), demonstrating that the response to *c-Myc* transduction substantially differs between juvenile and adult metaphyseal osteoblasts, and that *c-Myc* induction is not sufficient to induce proliferation of adult osteoblasts.

Remarkably, inhibition of Hedgehog signaling almost blocked the enhanced proliferation of *c-Myc*-induced juvenile osteoblasts (Fig. 5h), indicating that, as in normal metaphyseal osteoblasts, the Hedgehog signaling pathway is responsible for active proliferation of *c-Myc*-induced juvenile osteoblasts. This is also supported by the fact that, in both *Cdkn1a-* and *Osx*-*CreERT2* models, *c-Myc*-induced proliferation was not sustained and cells ceased proliferating at a similar time as that of normal osteoblasts (7 weeks of age), which was concurrent with maturation of the growth plate when IHH expression is diminished (Fig. 5i, and Supplementary Fig. 9a, c). Notably, at Day 28, although abnormal expansion of mGFP+ cells was not evident, the mGFP signal was detected in cells within the trabecular bone (Fig. 5i), suggesting that the expanded cells differentiate, at least in part, into osteocytes to generate trabecular bone. Our findings demonstrate that proliferation of *c-Myc*-induced metaphyseal osteoblasts depends on Hedgehog signaling, yet *c-Myc* activation alone is insufficient for sustained proliferation in *p21*+ juvenile osteoblasts to drive osteosarcoma development.

### p53 inactivation sustains *c-Myc*-induced growth

**Fig. 6:**
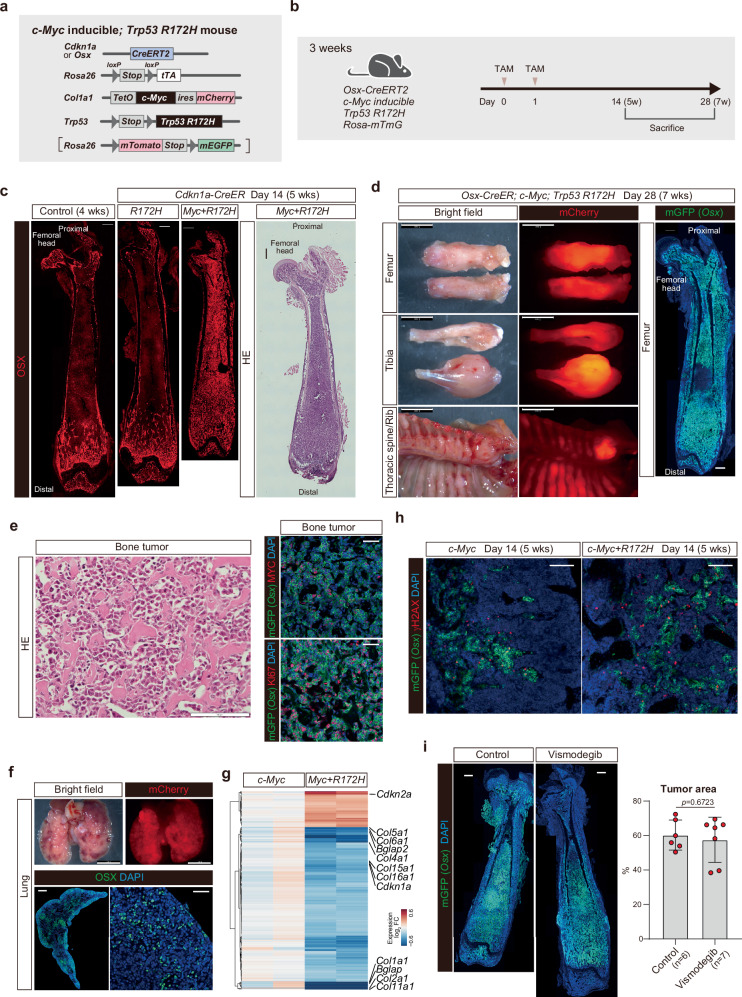
Inactivation of p53 confers sustained growth capacity in *c-Myc*-induced osteoblasts. **a** Schematic of the genetic construct used for simultaneous induction of p53 inactivation and *c-Myc* expression in osteoblasts. **b** Experimental protocol for combined induction of p53 inactivation and *c-Myc* expression. **c** Left: Immunofluorescence analysis of OSX expression in femurs from the *Cdkn1a-CreERT2* model. Combined induction of *c-Myc* and *Trp53R172H*, but not *Trp53R172H* alone, in *p21*+ cells results in selective expansion of OSX+ cells. Right: HE staining of femurs showing extensive expansion of abnormal osteoblasts throughout the femur by day 14. **d** Macroscopic images of long bones following co-induction of p53 inactivation and *c-Myc* expression in the *Osx-CreERT2* model. All long bones exhibit tumor formation. **e** Histological analysis of bone tumors from *c-Myc*; *Trp53R172H* mice in the *Osx-CreERT2* model. HE staining reveals expansion of dysplastic osteoblasts with frequent mitotic figures and osteoid formation, consistent with osteogenic osteosarcoma. Right: Active proliferation of the expanded osteoblast population. **f** Lung metastases derived from *Osx*+ osteoblasts. OSX+ cells are broadly disseminated throughout the lungs 28 days after co-induction of p53 inactivation and *c-Myc* expression. **g** Heatmap of differentially expressed genes in *c-Myc*-induced osteoblasts with p53 inactivation (adjusted *p* < 0.05). *Osx*-mGFP+ metaphyseal cells were collected 7 days after two consecutive tamoxifen treatments. Differentiation-associated genes are downregulated. Expression values are shown as log2 fold changes relative to the *c-Myc* alone group. **h** γH2AX expression is similarly detected in both *c-Myc* and *c-Myc*+*Trp53R172H* osteoblasts. **i** Left: Vismodegib treatment fails to suppress aberrant proliferation of *c-Myc*+*Trp53R172H* osteoblasts. Right: Quantification of tumor area. *n* indicates the number of mice analyzed. Source data are provided as a Source Data file. Data are shown as mean ± SD. Representative images are from at least three biologically independent mice. Statistical significance was determined by a two-sided unpaired Student’s t-test. The exact *P* value is indicated in the figure. Scale bars: **c** and **i**, 500 μm; **d**, left, 5 mm, right, 500 μm; **e**, left, 500 μm, right, 50 μm; **f**, upper, 5 mm, lower left, 500 μm, lower right, 50 μm; **h**, 50 μm.

Finally, given that the enhanced DDR and p53 activation were observed in *c-Myc*-induced juvenile osteoblasts (*c-Myc* osteoblasts), we introduced a dominant-negative mutation of the *Trp53* gene^31^ into *c-Myc*-induced metaphyseal cells using both the *Cdkn1a-CreERT2* and *Osx-CreERT2* models (Fig. 6a, b). Introduction of the *Trp53 R172H* mutation together with *c-Myc* induction (*c-Myc* + *R172H*) led to rapid osteoblast expansion with similar kinetics in both models (Fig. 6c, d, and Supplementary Fig. 11a). Notably, the abnormal proliferation of osteoblasts was sustained in adult mice (7 weeks of age), resulting in tumor development in all long bones with 100% penetrance (*n* = 10, Fig. 6d, e, and Supplementary Fig. 11b). Histological analyses revealed that the developed tumors were osteogenic osteosarcomas (Fig. 6e). Moreover, *c-Myc* + *R172H* osteoblasts metastasized to distant organs, including the lungs, the predominant site of metastasis in human osteosarcoma (Fig. 6f, and Supplementary Fig. 11c). RNA-seq analyses at Day 7 revealed that *p21* expression was reduced in *c-Myc* + *R172H* osteoblasts, consistent with *p21* being a transcriptional target of p53^18^ (Fig. 6g, and Supplementary Fig. 12a). By contrast, *Arf* expression was elevated in *c-Myc* osteoblasts and further augmented in *c-Myc* + *R172H* osteoblasts, presumably reflecting an adaptive response to abnormal cell-cycle progression (Fig. 6g, and Supplementary Fig. 12a). However, *p16* expression was not evident even in *c-Myc* osteoblasts (Supplementary Fig. 12a), suggesting that typical senescence is not involved in the growth arrest of *c-Myc*-induced cells^35^. *c-Myc* + *R172H* osteoblasts exhibited reduced expression of differentiation-related genes compared with *c-Myc* osteoblasts (Fig. 6g). Consistently, GO analysis and GSEA suggested that cell cycle progression was increased, apoptosis was suppressed, and differentiation was inhibited in *c-Myc* + *R172H* osteoblasts (Supplementary Fig. 12b, Table S1). Withdrawal of *c-Myc* expression after expansion of *c-Myc* + *R172H* osteoblasts abolished abnormal cells (Supplementary Fig. 12c), demonstrating that *c-Myc* is required for neoplastic proliferation of *c-Myc* + *R172H* osteoblasts. Expression of γH2AX was similarly observed in proliferating *c-Myc* + *R172H* osteoblasts and *c-Myc* osteoblasts (Fig. 6h), indicating a persistent DDR even after p53 inactivation. Remarkably, despite its potent inhibitory effect on proliferation of *c-Myc* osteoblasts, vismodegib almost failed to suppress proliferation of *c-Myc* + *R172H* osteoblasts in vivo (Fig. 6i), suggesting that the mitogenic signal was altered after p53 inactivation. Moreover, *c-Myc* induction in the presence of *Trp53 R172H* mutation led to osteosarcoma development even in adult mice (starting at 8 weeks of age) (Supplementary Fig. 12d), further supporting the notion that p53 inactivation elicits the Hedgehog-independent growth ability of *c-Myc*-induced osteoblasts.

## Discussion

IHH, one of the ligands of the Hedgehog signaling pathway, is expressed in cartilage tissue during embryonic development and postnatal growth^36–38^. It plays a crucial role in osteogenesis, especially in the formation of trabecular bones through endochondral ossification^38–43^. Extensive studies in embryonic and neonatal mouse models have shown that IHH expression in prehypertrophic chondrocytes, together with PTHrP expression in resting-zone chondrocytes, coordinates chondrocyte proliferation and differentiation, thereby regulating longitudinal bone growth^36,44–48^. However, our understanding of metaphyseal cells at the juvenile stage—, when osteosarcomas typically arise—, remains limited. This is partly because most metaphyseal phenotypes in mutant mouse models already manifest at the neonatal stage and partly due to the lack of markers for the juvenile stage, which hinders analyses at this stage^39–43^. Therefore, the proliferative dynamics and underlying molecular mechanisms of juvenile metaphyseal cells implicated in osteosarcoma development are yet to be fully elucidated.

Here, we demonstrate that Hedgehog signaling drives the proliferation of juvenile metaphyseal osteoblasts. scRNA-seq and immunofluorescence analyses revealed a spatiotemporal association between IHH expression in prehypertrophic chondrocytes at the growth plate and the emergence of *p21*+ osteoblasts in the metaphysis. Given their anatomical proximity and temporal correlation, our findings suggest that chondrocyte-derived IHH activates Hedgehog signaling and supports the proliferative capacity of juvenile metaphyseal osteoblasts. We also found that the *Gli1*-high multipotent progenitor cluster exhibited the highest *Gli2* expression, which is consistent with previous evidence that Hedgehog signaling maintains multipotent progenitors^21^. Notably, *Gli2* expression progressively decreased upon osteoblast specification and differentiation. Moreover, although *Gli2* expression was highest in multipotent progenitors, proliferative activity peaked in specified osteoblasts, resembling other tissues in which stem/progenitor cells divide infrequently while committed progenitors proliferate robustly before terminal differentiation^49^. These findings suggest that Hedgehog activity exerts context-dependent functions in the juvenile metaphyseal cells. Collectively, our findings delineate the sequential progression of osteoblast differentiation with distinct proliferative kinetics and support a model in which transient, spatially restricted IHH expression maintains metaphyseal homeostasis by regulating proliferation and differentiation of not only multipotent progenitors^21^ and chondrocytes^45^ but also osteoblast-lineage cells residing close to the growth plate. The observed negative correlation between *Gli1*/*Gli2* expression levels and the distance from IHH-expressing cells further highlights the role of IHH in orchestrating juvenile metaphyseal homeostasis. Although this study primarily focuses on metaphyseal osteoblasts, our data indicate that *c-Myc*-induced osteoblast proliferation in the diaphysis also depends on Hedgehog signaling. Considering the distance from the growth plate, it is unlikely that growth plate-derived IHH directly regulates this proliferation. Notably, scRNA sequencing identifying *Fgfr3*+ endosteal stem cells at the diaphysis^16^ showed that *Ihh*, but not *Shh* or *Dhh*, is expressed in *Fgfr3+* non-chondrocytes. Although *Ihh* expression levels in the diaphysis are substantially lower than in the metaphysis (Supplementary Fig. 12e), it is possible that IHH produced by these cells contributes to proliferation of *c-Myc*-induced juvenile osteoblasts at sites distant from the growth plate. Further studies, including genetic approaches targeting Hedgehog signaling in specific cell populations, will be required to clarify the precise cellular source and functional contribution of IHH in this context.

Importantly, we propose that this intrinsic regulation of juvenile osteoblast proliferation underlies the characteristic features of osteosarcoma development in the metaphysis at the juvenile stage. *c-Myc* expression induced robust proliferation only at a juvenile stage in a Hedgehog-dependent manner, but this effect was not sustained after growth plate maturation. In contrast, p53 inactivation enabled Hedgehog-independent proliferation and conferred metastatic potential in both juvenile and adult *c-Myc*-expressing osteoblasts. Given that *c-Myc* amplification is often an initiating event in osteosarcoma development, which could precede *TP53* mutations^50^, an activated proliferation, caused by *c-MYC* amplification, may predispose juvenile osteoblasts to acquire *TP53* mutations and progress toward osteosarcoma.

We show that immature osteoblasts exhibit DNA replication-associated DDR, leading to *p21* expression. Although DDR-related genes, especially *TP53* and to a lesser extent *ATM* and *BRCA2* are mutated in osteosarcomas^51^, the mechanistic link between DNA damage and tumor initiation remains to be fully elucidated. Using a unique mouse model, we demonstrated that *c-Myc*, a well-established inducer of replication stress^32^, enhances the DDR and triggers p53 activation in juvenile osteoblasts through their intrinsic features. Remarkably, *c-Myc*-expressing osteosarcomas that developed following p53 inactivation retained robust γH2AX expression, indicating that DDR persists even after malignant transformation. These findings suggest that p53-mediated checkpoint activation functions as a protective mechanism that limits the propagation of stressed proliferating osteoblasts, whereas loss of p53 permits continued proliferation under replication stress-associated conditions, thereby promoting osteosarcoma development. These results further suggest that activation of p53, rather than the DDR per se, plays a central role in tumor suppression. Of note, although *Rb* loss markedly accelerates osteosarcoma development in the *Trp53*-deficient mice, it is insufficient on its own^12,13^. We propose that aberrant cell-cycle progression caused by RB inactivation amplifies replication stress, leading to p53 activation and thereby phenocopying the effects of *c-Myc* induction. These mechanisms may also explain why the vast majority of human osteosarcomas harbor *TP53* mutations in combination with genetic alterations that promote cell-cycle progression, such as *c-MYC* amplification and *RB* loss.

Given the widespread occurrence of enhanced proliferation and *TP53* mutations across many cancer types, our findings may extend beyond osteosarcoma and offer critical insights into the causal relationship between early tumorigenesis and DNA damage-induced p53 activation. Based on our results, we propose that early proliferative lesions experience sustained replication stress, which in turn triggers p53 activation to suppress malignant transformation. Supporting this concept, the acute p53 response to radiation is not essential for lymphoma suppression, whereas delayed activation of p53 provides protective effects^52–54^.

In conclusion, we propose that intrinsic DDR to replication-associated stress, together with the spatiotemporal restriction of mitogenic signals, safeguards against oncogenic transformation in metaphyseal osteoblasts. In juvenile osteoblasts, DNA replication-associated stress responses and restricted IHH expression may limit the persistence of aberrantly proliferating cells, thereby underscoring the requirement for aberrant cell-cycle progression and p53 inactivation in osteosarcoma development. These mechanisms may underlie the juvenile onset, metaphyseal localization, and characteristic genetic alterations of human osteosarcomas.

## Methods

### Ethics statement

All experiments complied with all relevant ethical regulations. All animal experiments were approved by the Animal Experiment Committees of the Institute of Medical Science and the Graduate School of Medicine, The University of Tokyo, and were conducted in accordance with institutional guidelines. The use of human tissue samples was approved by the Ethics Committee of the Graduate School of Medicine and Faculty of Medicine, The University of Tokyo, and informed consent was obtained from participants or their legal guardians.

### Vector construction

#### Osx-CreERT2 vector

A cDNA fragment encoding *CreERT2-pA-PGK-Bsd-pA* (3.8 kb) flanked by 70-bp (5′) and 90-bp (3′) homology arms was generated using KAPA HiFi HotStart ReadyMix (KAPA Biosystems). This fragment was recombined at the first ATG in exon 1 of the *Osx* BAC (BACPAC Resources Center) using the Red/ET BAC recombination system. The resulting *Osx-CreERT2-pA-PGK-Bsd-pA* cassette, with 1.0-kb (5′) and 1.5-kb (3′) homology arms, was retrieved and used as a targeting vector.

#### p21-IRES-CreERT2 vector

A cDNA fragment encoding *IRES-CreERT2-pA-PGK-Bsd-pA* (4.3 kb), flanked by 20-bp homology arms, was generated using KAPA HiFi HotStart ReadyMix (KAPA Biosystems). This fragment was recombined between the stop codon of exon 3 and the 3′ UTR of the *Cdkn1a* BAC (BACPAC Resources Center) using the Red/ET BAC recombination system. The resulting *p21-IRES-CreERT2-pA-PGK-Bsd-pA* cassette, with 1.0-kb (5′) and 1.6-kb (3′) homology arms, was retrieved and used as a targeting vector.

### *Osx-CreERT2* knock-in using CRISPR/Cas9

A guide RNA (gRNA) targeting the *Osx* locus was designed using CRISPRdirect (http://crispr.dbcls.jp/). The gRNA sequence was: CCCGGTCCCCAGCTCGAGGA. The gRNA oligonucleotide was cloned into the pX330-U6-Chimeric_BB-CBh-hSpCas9 plasmid (Addgene, #42230).

### Homologous recombination in embryonic stem cells (ESCs)

V6.5 mouse ESCs were used for the generation of genetically modified mouse lines. The targeted ESC clones were validated by PCR-based genotyping and sequencing of the targeted genomic loci before blastocyst injection. ESC cultures were confirmed to be negative for mycoplasma contamination.

Twenty micrograms of each targeting vector were linearized using restriction enzymes (ZraI for the *Osx-CreERT2* vector and MfeI-HF for the *p21-IRES-CreERT2* vector). All enzymes were purchased from New England Biolabs. Following overnight digestion at 37 °C, the DNA was purified by ethanol precipitation, and the resulting pellets were resuspended in 100 μL of 25 mM HEPES buffer (Gibco).

V6.5 ESCs were dissociated using 0.25% trypsin (Nacalai Tesque) and resuspended in 500 μL of high-glucose DMEM (Nacalai Tesque) supplemented with 25 mM HEPES^55^. For *Osx-CreERT2* targeting, the linearized vector and 5 μg of a Cas9/gRNA complex, which comprised the *Osx*-targeting gRNA and the pX330-U6-Chimeric_BB-CBh-hSpCas9 plasmid, were mixed with 4.0 × 10^6^ ESCs. The mixture was transferred to a Gene Pulser cuvette (Bio-Rad) and electroporated using the Gene Pulser Xcell system (Bio-Rad) under the following conditions: 550 V for 600 ms, twice.

For *p21-IRES-CreERT2* targeting, the linearized vector alone was mixed with 4.0 × 10^6^ ESCs and electroporated under the same conditions. After electroporation, 2.0 × 10^6^ cells were plated onto mitotically inactivated mouse embryonic fibroblasts in each of two 6-cm culture dishes and maintained at 37 °C in 5% CO_2_ in ESC culture medium [KnockOut DMEM (Gibco) supplemented with 2 mM L-glutamine (Nacalai Tesque), 1× non-essential amino acids (Nacalai Tesque), 100 U/mL penicillin, 100 μg/mL streptomycin (Nacalai Tesque), 15% fetal bovine serum (Gibco), 0.11 mM 2-mercaptoethanol (Gibco), and 1000 U/mL human leukemia inhibitory factor (Wako)].

Twenty-four h after electroporation, antibiotic selection was initiated with 15 μg/mL blasticidin S (Funakoshi). After 1 week of selection, surviving ESC colonies were picked and expanded to establish ESC lines.

### Mice experiments

All animal care and procedures, including those involving tumor-bearing mice, were conducted in accordance with institutional guidelines. All animal experiments were performed with efforts to minimize suffering. In vivo experiments were ended according to the planned experimental schedule unless mice reached humane endpoints earlier. Humane endpoints included rapid body weight loss (>20% within a few days), severe respiratory distress, marked deterioration in general condition, or reduced mobility. In tumor-bearing mice, bleeding from tumors or excessive tumor growth, defined as tumor burden exceeding approximately 10% of body weight, was also used as a humane endpoint. Mice reaching any of these criteria were euthanized immediately. 

### Blastocyst collection and microinjection

For blastocyst preparation, 8-week-old ICR female mice (Japan SLC) were intraperitoneally injected with 7.5 U of serotropin (ASKA Animal Health). Forty-eight h later, they received an additional intraperitoneal injection of 7.5 U of gonadotropin (ASKA Pharmaceutical) and were subsequently mated with ICR male mice (Japan SLC). Vaginal plugs were checked the following morning. Two days post-coitum, two-cell-stage embryos were collected via oviduct perfusion using M2 medium (Sigma) and cultured in KSOM medium to allow development to the blastocyst stage.

For microinjection, ESCs were dissociated into single cells by trypsinization, followed by 15 cycles of pipetting. The resulting cell suspension was plated onto a gelatin-coated 10-cm dish containing 10 mL of ESC medium and incubated for 30 min to allow selective attachment of mouse embryonic fibroblasts. Subsequently, 3 mL of the supernatant containing ESCs was collected. Three to five ESCs were injected into each ICR blastocyst using an OLYMPUS IX71 microscope. A total of 20–25 injected blastocysts were transferred into the uterus of each pseudopregnant ICR female mouse (Japan SLC).

### Generation of *p21*-reporter *Atm* knockout chimeras

An *mTmG* reporter allele (Addgene #17787) was introduced into the *Rosa26* locus of the established ESC lines to generate *p21-IRES-CreERT2*; *Rosa26-mTmG* ESCs. Subsequently, CRISPR/Cas9-mediated genome editing was performed of these ESC lines to generate *p21-IRES-CreERT2*; *Rosa26-mTmG*; *Atm* KO ESCs^56^. Two gRNAs flanking exon 5, which contains the protein-coding region, were designed using CRISPOR2^57^. *Atm*-targeting Cas9 ribonucleoprotein complexes, which comprised two gRNAs targeting the *Atm* locus (IDT) and Alt-R S.p. Cas9 Nuclease V3 (IDT) were electroporated into *p21-IRES-CreERT2*; *Rosa26-mTmG* ESCs using the Neon Transfection System (Thermo Fisher). The sequences of the gRNAs targeting the *Atm* locus were as follows: gRNA1, GATGTATTCTATTAAACTCA and gRNA2, TGGTACAATATGAAACCAAG. After electroporation, the ESCs were passaged once and then picked and expanded to establish ESC lines. To confirm genome editing, the *Atm* genomic locus was amplified by PCR using a primer set flanking exon 5, and the amplicons were sequenced using the PCR enzyme KOD FX Neo (TOYOBO). The following primers were used for genotyping: Forward, GTTATATACAAACAGAGCAAACTGTGAAGCC and Reverse, CCTCAGACCCTTGTGTCACTATCTC. The established *Atm* KO ESC line was then used for blastocyst injection to generate chimeric mice.

### Mouse strains and husbandry

*Col1a1::TetO-cMyc-IRES-mCherry* knock-in, *Trp53-LSL-R172H* knock-in, and *Rosa26-LSL-tTA* knock-in mice have been described previously^31,33,34,58^. All mice used in this study were maintained on a mixed C57BL/6–129×1/Sv genetic background, unless otherwise indicated. The number and age of animals used in each experiment are indicated in the figure legends and Source Data file. Male mice were used for RNA-seq and scRNA-seq experiments. For other experiments, both male and female mice were used, but sex was not considered as a biological variable in the study design or statistical analyses. For germline transmission, 8-week-old male chimeric mice were crossed with 8-week-old C57BL/6 female mice (Japan SLC) to generate transgenic offspring. Wild-type bone samples were collected from C57BL/6 J mice at the indicated ages. *Rosa26-mTmG* reporter mice were obtained from The Jackson Laboratory. All mice were housed under specific pathogen-free conditions in a room with a 12-hour light/dark cycle, at 20–24 °C and 45–65% humidity, with ad libitum access to food and water.

### Mice genotyping

Tail tips were collected from 3-week-old mice and lysed in 100 μL of DNA lysis buffer (100 mM Tris-HCl, 5 mM EDTA, 0.2% SDS, 200 mM NaCl, and 1% proteinase K) at 65 °C. After incubation, 50 μL of saturated NaCl solution was added, thoroughly mixed, and centrifuged at 14,000 × *g* for 15 min at 4 °C. Then, 100 μL of the resulting supernatant was transferred to 100 μL of 100% ethanol and mixed to precipitate genomic DNA. The sample was centrifuged again at 14,000 × *g* for 15 min at 4 °C. After discarding the supernatant, the DNA pellet was air-dried completely and resuspended in 100 μL of Tris-EDTA buffer. Genotyping was performed by PCR using GoTaq Master Mix (Promega). Primer sequences used for genotyping are listed in Table S2.

### Tamoxifen treatment

Tamoxifen (Sigma) was dissolved in corn oil (Wako) at a concentration of 20 mg/mL. *p21* reporter, *Osx* reporter, and *Atm* KO mice received intraperitoneal injections of tamoxifen at a dose of 100 mg/kg body weight at the indicated time points. For the analysis of embryonic femurs, pregnant p21 reporter mice received a single intraperitoneal injection of tamoxifen (50 mg/kg body weight) at embryonic day 13.5 (E13.5). To induce *Trp53-R172H* in *p21*+ cells, 4-week-old mice were injected intraperitoneally with tamoxifen at a dose of 100 or 200 mg/kg body weight. For the induction of *c-Myc*, or combined induction of *c-Myc* and *Trp53-R172H*, *p21* and *Osx* reporter mice harboring Tet-OFF inducible transgenes received intraperitoneal tamoxifen at 1 mg/mouse at 3 weeks of age and 2 mg/mouse at 8 weeks of age.

### Doxycycline and tetracycline administration

To prevent leaky transgene expression, tet-OFF inducible mice or pregnant females were administered doxycycline hyclate (0.5 mg/mL; Sigma) or tetracycline hydrochloride (2–4 mg/mL; Wako) in drinking water supplemented with 10–20 mg/mL sucrose (Nacalai Tesque) until 2 weeks (doxycycline) or 1 week (tetracycline) prior to analysis, respectively^34^.

### EdU labeling

To label proliferating cells, mice were intraperitoneally injected with 500 μg of EdU (Invitrogen) and euthanized 3 h later. Bones were immediately harvested and processed as described below. Frozen bone sections were stained for EdU using a Click-iT EdU Imaging Kit (Invitrogen), following the manufacturer’s instructions.

### Doxorubicin treatment

Six-week-old *p21* reporter mice received intraperitoneal injections of doxorubicin (10 mg/kg) at the indicated time points. To evaluate age-dependent responses to DNA damage, three- and eight-week-old C57BL/6 J wild-type mice received intraperitoneal injections of doxorubicin (5 mg/kg) and euthanized 48 h later. Bones were immediately harvested and processed as described below.

### Vismodegib administration

Vismodegib (SelleckChem, Cat. No. S1082) was formulated in 0.5% (w/v) methylcellulose (Wako) and 0.2% (v/v) Tween- 80 (SelleckChem) for oral administration^29^. Briefly, 50 mg of vismodegib was dissolved in 5 mL of vehicle to prepare a 10 mg/mL stock solution. Mice received daily oral gavage of vismodegib suspension or vehicle at a dose of 100 mg/kg body weight.

### Preparation of frozen tissue sections

Freshly isolated long bones (femurs or tibias), lungs, and livers were collected and fixed in 4% paraformaldehyde (Wako) at 4 °C for 16–24 h with gentle rocking on a horizontal shaker. Bone samples were decalcified in 0.5 M EDTA (pH 8.0; Nacalai Tesque) at 4 °C for 48 h to 7 days with gentle rocking. Following decalcification, tissues were cryoprotected in 30% sucrose at 4 °C for 2 days under the same conditions. Samples were then embedded in optimal cutting temperature compound (Sakura Finetek) and cryosectioned using a Leica CM2050 or CM1850 cryostat.

Bone sections were cut at 60 μm thickness for immunofluorescence staining and at 15–20 μm thickness for hematoxylin and eosin (HE) staining. Visceral organs were sectioned at 4 μm thickness for immunofluorescence. Sections were prepared using low-profile tungsten carbide blades (16 cm; Cat. No. 14021604206, Leica Biosystems).

### Immunofluorescence staining of frozen sections

Frozen sections were air-dried at room temperature, rehydrated in phosphate-buffered saline (PBS), and permeabilized with 0.3% Triton X-100 prepared in PBS for 30 min. Sections were then blocked with Protein Block Serum-Free Ready-To-Use (Dako) at room temperature for 30 min. Primary antibodies were diluted in IMMUNO SHOT (Cosmo Bio) and applied to the sections, which were incubated overnight at 4 °C in a humidified, light-protected chamber. The following primary antibodies were used: rabbit anti-p-RPA (pS4/S8) (1:400; ab87277, Abcam), rat anti-EMCN (1:200; SC-65495, Santa Cruz), rabbit anti-OSX (1:500–1:750; ab209484, Abcam), rabbit anti-p21 (1:500–1:750; ab188224, Abcam), rabbit anti-KI67 (1:200; ab16667, Abcam), rabbit anti-c-MYC (1:200; ab32072, Abcam), rabbit anti-RUNX2 (1:500–1:750; ab192256, Abcam), rabbit anti-IHH (1:500–1:1000; ab39634, Abcam), rabbit anti-Fabp4 (1:200; ab13979, Abcam), rabbit anti-phospho-Histone H2A.X (Ser139) (1:500–1:750; 9718, Cell Signaling), rabbit anti-cleaved caspase-3 (Asp175) (1:200; 9661, Cell Signaling), rabbit anti-phospho-p53 (Ser15) (1:200–1:500; 9284, Cell Signaling), rabbit anti-integrin β3 (1:200; 4702, Cell Signaling), and rabbit anti-COL1A1 (1:200; CL50151AP, Cedarlene). After washing three times with PBS, sections were incubated overnight at 4 °C with secondary antibodies diluted 1:500 in PBS in the same chamber. The following secondary antibodies were used: Alexa Fluor 647-conjugated donkey anti-rabbit IgG (A31573, Invitrogen), Alexa Fluor 488-conjugated goat anti-rabbit IgG (A11034, Invitrogen), and Alexa Fluor 647-conjugated goat anti-rat IgG (A21247, Invitrogen). After incubation, sections were washed three times with PBS and mounted using ProLong Gold Antifade Reagent (P36930, Invitrogen). Nuclei were counterstained with DAPI (340-07971, DOJINDO). For immunostaining with rabbit anti-p-RPA (pS4/S8), Tris-buffered saline (TBS) was used instead of PBS. In addition, Halt™ Protease and Phosphatase Inhibitor Cocktail, EDTA-free (100×) (78440, Thermo Fisher), was added at a 1:100 dilution throughout the permeabilization, blocking, and antibody incubation steps. Tumor areas were evaluated in DAPI-stained bone sections using Fiji (ImageJ, NIH). We took advantage of the characteristic that hematopoietic cells in the bone marrow exhibit strong DAPI fluorescence, whereas tumor cells display relatively weaker staining. To quantify tumor burden, we subtracted the area of strong DAPI signals from the total DAPI-positive area within the bone, with the resulting value considered as the tumor area.

### Confocal microscopy

Fluorescent images were acquired using a confocal laser scanning microscope (LSM900, Zeiss). Image acquisition and basic processing were performed using ZEN 3 (Blue Edition) software (Zeiss), and final adjustments were made using Adobe^33,34^ trator (v30.5.1, Adobe Systems). Representative images are shown in the figures. Cells of interest were quantified with Fiji (ImageJ, NIH) in high-resolution images.

### Preparation of paraffin-embedded tissue sections

Dissected tissue samples were fixed in 4% paraformaldehyde (Wako) overnight at room temperature. The following day, visceral organs were processed, whereas bones were decalcified for approximately 1 month in 0.5 M EDTA (pH 8.0; Nacalai Tesque) at 4 °C with gentle rocking on a horizontal shaker. After decalcification, tissues were dehydrated in 70% ethanol for several days and then embedded in paraffin using a HistoCore PEARL tissue processor (Leica Biosystems). Paraffin-embedded blocks were sectioned at a thickness of 4–6 μm.

### HE staining

For paraffin-embedded tissue sections, slides were baked at 65 °C for 1 h and then deparaffinized by immersion in xylene (three times for 5 min). For frozen sections, slides were rehydrated in PBS. Both types of sections were then immersed in 100% ethanol (three times for 1.5–2 min, total of 5 min) to achieve hydrophilization. After washing with distilled water for several minutes, sections were stained with hematoxylin for 8 min followed by eosin for 5 min. Slides were then dehydrated in 100% ethanol (three times), cleared in xylene (three times), and mounted with coverslips. HE-stained sections were imaged using a BX41 microscope (Olympus). For the merged image, sections were photographed using a BZ-X710 fluorescence microscope (KEYENCE).

### Immunohistochemistry of paraffin-embedded tissue sections

Paraffin sections were baked at 65 °C for 1 h, deparaffinized by immersion in xylene (three times for 5 min), and rehydrated in 100% ethanol (three times for 1.5–2 min, total of 5 min). After rinsing in distilled water for several min, antigen retrieval was performed by immersing sections in epitope retrieval buffer (DAKO) and autoclaving at 105 °C for 10 min. Sections were then rinsed in 1× PBS and incubated overnight at 4 °C with 100 μL of a primary antibody diluted in IMMUNO SHOT (Cosmo Bio). The following primary antibodies were used: rabbit anti-phospho-Histone H2A.X (Ser139) (1:500–1:750; Cell Signaling, 9718) and rabbit anti-p21 (1:500–1:750; Abcam, ab188224). For human samples, mouse anti-p21 (1:200; Santa Cruz, sc-6246), rabbit anti-KI67 (1:200; ab16667, Abcam), and rabbit anti-OSX (1:500; ab209484, Abcam) were used. After washing twice with PBS (5 min each), sections were incubated for 30 min at room temperature with horseradish peroxidase-conjugated secondary antibodies (Histofine, Nichirei), followed by development with DAB (Nichirei). Slides were washed again with PBS (two times for 5 min), counterstained with Mayer’s hematoxylin for 10–30 seconds, and rinsed in water for 10 min. Finally, sections were dehydrated in 100% ethanol (three times), cleared in xylene (three times), and mounted with MOUNT-QUICK (DAIDO SANGYO, Japan). Images were acquired using a BX41 microscope (Olympus).

### Paraffin-embedded human tissue samples

Paraffin-embedded human distal femur tissue samples from five participants were used with the approval of the Research Ethics Committee of the Graduate School of Medicine and Faculty of Medicine, The University of Tokyo. Informed consent was obtained from each participant or their legal guardian. The samples were obtained from juvenile participants, and the age range and sex distribution are provided in the Source Data file. Participants received no compensation. Sex was determined from clinical records. Sex and gender were not considered as biological variables in the study design or statistical analyses because the human samples were used only to confirm p21 expression in juvenile metaphyseal osteoblasts by immunohistochemistry, and no sex- or gender-based comparison was performed.

### Cell preparation for RNA-seq of mGFP+ osteoblasts

mGFP+ osteoblasts were isolated from 3- and 8-week-old *Osx-CreERT2*; *mTmG* mice for RNA-seq analysis. Two femurs and two tibias were dissected from each mouse, and surrounding soft tissues were carefully removed. For control samples, femurs and tibias from two male mice were pooled per replicate. For *c-Myc*-induced and *c-Myc* + *Trp53-R172H* osteoblasts, mGFP+ cells were isolated from *Osx-CreERT*2; *mTmG*; *LSL-tTA*; *Col1a1*::*TetO–c-Myc* and *Osx-CreERT2*; *mTmG*; *LSL-tTA*; *Col1a1*::*TetO–c-Myc*; *Trp53-LSL-R172H* mice, respectively (both male and female), 7 days after tamoxifen administration.

### Bone dissociation to collect mGFP+ osteoblasts

To isolate cells specifically from the metaphyseal region, the epiphysis was carefully removed at the midpoint of the growth plate using forceps. Residual chondrocytes from the growth plate were thoroughly removed under a stereomicroscope. The diaphysis was then trimmed approximately 2 mm (for 3- and 8-week-old *Osx-CreERT2*; *mTmG* mice) or 5 mm (for 5-week-old *Osx-CreERT2*; *mTmG*; *LSL-tTA*; *Col1a1*::*TetO–c-Myc* and *Osx-CreERT2*; *mTmG*; *LSL-tTA*; *Col1a1*::*TetO–c-Myc*; *Trp53-LSL-R172H* mice) distal to the growth plate.

The harvested metaphyseal regions from distal femurs and proximal tibias were transferred to digestion buffer comprising M199 with Hanks’ salts (Gibco) containing 250 U/mL type I collagenase (Worthington), 250 U/mL type II collagenase (Worthington), 100 U/mL DNase I (1 mg/mL; Roche), 1 mg/mL poloxamer 188 (Sigma), 1 mg/mL BSA (Fujifilm), and 20 mM HEPES (Nacalai Tesque). The bones were placed in a mortar with 1 mL of digestion buffer and crushed using a pestle for at least 1 min. The supernatant was collected in a 15 mL conical tube. This process was repeated until the bones appeared completely white (typically three times or more).

The remaining bone fragments were pooled into the same tube and supplemented with digestion buffer to a final volume of 5 mL per sample. Samples were incubated at 37 °C for 30 min at 210 rpm. Following digestion, the supernatant was filtered through a 70 μm cell strainer into a 50 mL tube containing 5–10 mL of ice-cold cell suspension buffer [Ca^2+^/Mg^2+^-free PBS (Nacalai Tesque) containing 2% fetal bovine serum, 1 mg/mL poloxamer 188, and 100 U/mL penicillin/streptomycin (Gibco)].

Bone fragments were further digested with 3–5 mL of prewarmed digestion buffer, and the crushing–filtration process was repeated 4–5 times. Red blood cells were lysed using ACK lysis buffer (Gibco). Bone chips were removed, and single-cell suspensions were obtained by filtering through a 40 μm strainer for subsequent magnetic-activated cell sorting (MACS) preparation.

### MACS and fluorescence-activated cell sorting (FACS)

MACS was performed using LD columns (Miltenyi Biotec, Cat. No. 130-042-901) to deplete hematopoietic and erythroid cells with CD45 microbeads (Cat. No. 130-052-301) and TER119 microbeads (Cat. No. 130-049-901), following the manufacturer’s instructions. Following MACS, single-cell suspensions were incubated on ice and in the dark for 30 min with the following fluorophore-conjugated antibodies (1:200 dilution; Invitrogen/eBioscience), prepared in cell suspension buffer: eFluor450-conjugated anti-CD31 (clone 390; Cat. No. 48-0311-82), eFluor450-conjugated anti-CD45 (clone 30F-11; Cat. No. 48-0451-82), and eFluor450-conjugated anti-TER119 (clone TER119; Cat. No. 48-5921-82).

FACS was performed using the FACS Melody system (BD Biosciences). mGFP+ cells were isolated from the CD31⁻CD45⁻TER119⁻ fraction after dead cells were excluded using 7-AAD (1:200; BioLegend, Cat. No. 420403). Sorted cells were immediately collected in Buffer RLT Plus (Qiagen) and stored at −80 °C until RNA extraction.

### Library preparation for RNA-seq

Total RNA was extracted using an RNeasy Plus Micro Kit (Qiagen) according to the manufacturer’s protocol. RNA concentration and purity were assessed using a NanoDrop 2000c spectrophotometer (Thermo Fisher), and RNA quantity was further confirmed using a Qubit fluorometer (Thermo Fisher). Samples with an RNA integrity number (RIN) ≥ 8, as determined by the Bioanalyzer (Agilent Technologies), were used for library preparation. RNA-seq libraries were generated from 200 ng of high-quality RNA using an NEBNext Ultra II Directional RNA Library Prep Kit for Illumina (New England Biolabs). The number of PCR amplification cycles was minimized to prevent amplification bias. Libraries were sequenced on a NextSeq 550 platform (Illumina) using 86-bp single-end reads.

### RNA-seq data analyses

The sequenced reads were mapped to the mm10 mouse reference genome using STAR v. 2.7.11a^59^ with the GENCODE vM25 annotation gtf file after trimming adaptor sequences and low-quality bases using cutadapt-4.6^60^. Uniquely mapped reads were summarized at the gene level using HTSeq-count v. 2.0.5^61^, with the GENCODE vM23 annotation file and differentially expressed genes (DEGs) were identified by calculating fold changes and adjusted-pvalues using DESeq2 v. 1.42.0^62^. Motif analyses were performed using HOMER version 4.11^63^ with the option -start −400 and -end 100. The Integrative Genomics Viewer (IGV, v2.6.2)^64^ was used to visualize sequencing reads.

### Cell preparation for scRNA-seq

For scRNA-seq, 3-week-old *p21* reporter mice and 3- or 8-week-old C57BL/6 J wild-type mice were used. *p21* reporter mice received a single intraperitoneal injection of tamoxifen (1 mg/mouse) 48 h prior to analysis. The diaphysis was trimmed approximately 2 mm distal to the growth plate, and metaphyseal regions of distal femurs and proximal tibias were collected in digestion buffer. Following bone dissociation as described above, FACS was performed using the FACS Melody system (BD Biosciences). CD31/CD45/TER119-negative cells were sorted after dead cells were excluded using 7-AAD (1:200; BioLegend, 420403). Sorted cells were immediately collected in ice-cold cell suspension buffer and loaded onto a Chromium Controller (10X Genomics). Single-cell libraries were prepared using Chromium Next GEM Single Cell 3′ Reagent Kits v3.1 (10X Genomics) and sequenced on a NovaSeq 6000 system (Illumina) using paired-end sequencing.

### Analysis of scRNA-seq data

The sequenced reads for scRNA-seq data were mapped and quantified using Cell Ranger pipelines (v. 7.2.0., 10X Genomics) against the reference genome (mm10), with --include-introns = false option. The raw unique molecular identifier counts data were imported into the Seurat package (v. 5.0.1)^65^. Cells with nfeature >1,500, nCount between 5,000 and 75,000, and low mitochondrial gene expression (<20%) were further analysed. A putative doublet cluster was removed by Doublet Finder (v2.0.4)^66^. The Seurat package SCTransform function was used to normalized the data. Each subset of data was integrated with the Seurat package IntegrateLayers function with anchor-based CCA integration. Uniform manifold approximation and projection (UMAP) analysis and clustering were performed using the Seurat RunUMAP function with the parameter dims = 1:50, and the FindClusters function with resolution values of 0.03 for Supplementary Fig. 5, 0.04 for Fig. 1f-j, 0.4 for Fig. 4e, Supplementary Fig. 7a-b and 0.5 for Fig. 4a-d, Supplementary Fig. 6. The UMAP analysis and clustering were performed using the Seurat RunUMAP function. The cell type was defined according to expression of selected markers. To determine the cell cycle phases, cell cycle scores were calculated based on the scoring strategy as described previously^67^. RNA velocity was analyzed using the Python package scVelo (v0.3.0. 2). The bam files generated with CellRanger were preprocessed with Velocyto (v0.17.17), and the RNA velocity estimated with scVelo dynamical model was visualized on UMAP embedding from the Seurat object.

### RNA extraction from tissues

Femurs (*n* = 2 per mouse) and tibiae (*n* = 2 per mouse) were harvested from 3- and 8-week-old C57BL/6 J mice. After removal of the articular cartilage, the metaphyseal regions (MP) of the distal femur and proximal tibia were rapidly dissected, collected into 1.5 mL tubes, and immediately snap-frozen in liquid nitrogen. For diaphyseal samples, bone marrow was flushed out with PBS, and the remaining cortical bone was collected into 1.5 mL tubes and immediately snap-frozen in liquid nitrogen. Frozen samples were pulverized using a mortar and pestle under liquid nitrogen. Subsequently, the powdered tissues were transferred into 1 mL of Sepazol (Nacalai Tesque) and homogenized using a sonicator (Qsonica) for a total of 30 seconds on ice. After homogenization, the samples were incubated at room temperature for 5 min. Subsequently, 200 μL of chloroform (Wako) was added, followed by vigorous mixing by inversion and vortexing. The mixtures were then incubated at room temperature for 3 min and centrifuged at 4 °C and 12,000 × *g* for 15 min. The aqueous phase (500 μL) was carefully collected and mixed with an equal volume (500 μL) of 70% ethanol. The mixtures were applied to an RNeasy MinElute spin column (Qiagen), and RNA was purified according to the manufacturer’s instructions.

### cDNA synthesis and quantitative PCR (qPCR) analysis

Total RNA was isolated using the RNeasy Micro (Qiagen). Five-hundred nanograms of RNA was reverse-transcribed into cDNA by using the PrimeScript RT Reagent Kit (Takara Bio). The quantitative real-time PCR analysis was performed using the GoTaq qPCR Master Mix and CXR Reference Dye (Promega) on a StepOnePlus Real-Time PCR system (Thermo Fisher Scientific). Transcript levels were normalized against the corresponding level of Gapdh. Experiments were performed in biological triplicate. The primers used are shown in Table S2.

### Western blot analysis

Cultured *p21-IRES-CreERT2*; *Rosa26-mTmG*; *Atm* KO ESCs were harvested in 500 μL of RIPA lysis buffer [10 mM Tris-HCl (pH 8.0), 150 mM NaCl, 1% Triton X-100, 1% sodium deoxycholate, and 0.1% SDS] supplemented with 0.5% protease inhibitor cocktail, 1% DTT, and 1× phosphatase inhibitor (Nacalai Tesque) on ice. Twenty micrograms of denatured protein was loaded per lane on Any kD™ Mini-PROTEAN® TGX™ Precast Protein Gels (Bio-Rad, Cat# 4569033). After electrophoresis, proteins were transferred to GeneScreen Plus membranes (Revvity). Membranes were incubated overnight at 4 °C with primary antibodies diluted in blocking buffer (2% skim milk prepared in TBST). The following primary antibodies were used: mouse monoclonal anti-ATM [GeneTex (2C1), Cat# GTX70103, 1:1000] and mouse monoclonal anti-GAPDH (Invitrogen, Cat# AM4300, 1:1000). After washing with TBST, membranes were incubated with secondary antibodies in blocking buffer for 1 h at room temperature. The secondary antibody used was horseradish peroxidase-conjugated sheep anti-mouse IgG (Cytiva, Cat# NA931, 1:1000). Detection was performed using Pierce ECL Plus Western Blotting Substrate (Thermo Scientific), and signals were visualized using an Amersham Imager 680 (Cytiva).

### Quantification of immunofluorescence staining

Quantification of immunofluorescence-positive cells was performed using Fiji software (v1.54). For each condition, at least three mice were analyzed, and at least three randomly selected fields per mouse were quantified. Values from multiple fields were averaged to generate a single biological replicate value for each mouse. Positive cells were identified by threshold-based image analysis in Fiji. Data are presented as mean ± SD.

### Statistics and reproducibility

All statistical parameters, including sample size, statistical tests and exact *p*-values, are described in the figures, figure legends or Source Data file. No statistical method was used to predetermine sample size. Sample sizes were chosen based on previous studies and standard practice in the field. No data were excluded from the analyses, except for cells excluded from scRNA-seq analyses according to the quality-control criteria described above. Statistical analyses were performed using Prism 8 software (GraphPad). For scRNA-seq analyses, Pearson’s correlation coefficients and P values were calculated using the cor.test function in R (version 4.3.2). Data are presented as the mean ± standard deviation (SD), unless otherwise stated. Mice were randomly assigned to experimental groups whenever applicable. The investigators were not blinded to allocation during experiments or outcome assessment. The reproducibility of representative images was confirmed in a minimum of three biologically independent samples, unless otherwise stated in the figure legends.

### Schematic illustrations

Schematic illustrations in Figs. 2a, 5b, 6b and Supplementary Figs. 1b, e, 3b and 8b were created by the authors using Adobe Illustrator.

### Reporting summary

Further information on research design is available in the Nature Portfolio Reporting Summary linked to this article.

## Supplementary information

Supplementary Information

Reporting Summary

Transparent Peer Review file

## Source data

Source Data file

## Data Availability

RNA-seq and scRNA-seq data have been deposited in the Gene Expression Omnibus under accession codes GSE297565 and GSE333438. Source data are provided with this paper. All other data supporting the findings of this study are available within the article, Supplementary Information and Source Data files. Source data are provided with this paper.

## Code availability

No custom code was used in this study.
