# Supplementary material for: Inherent tissue homeostasis of the juvenile metaphysis provides a foundation for osteosarcoma development
Source: Nat Commun. 2026 Jun 26;17:6241. doi: 10.1038/s41467-026-74929-6 (PMC13373234; doi:10.1038/s41467-026-74929-6)
Supplement: Supplementary file 1 — Supplementary Information [file 41467_2026_74929_MOESM1_ESM.pdf]

Inventory of Supplementary Information

Inherent tissue homeostasis of the juvenile metaphysis provides a foundation for osteosarcoma development

Saito et al.

This file contains:

Supplementary Figures

Supplementary Fig. 1
Supplementary Fig. 2
Supplementary Fig. 3
Supplementary Fig. 4
Supplementary Fig. 5
Supplementary Fig. 6
Supplementary Fig. 7
Supplementary Fig. 8
Supplementary Fig. 9
Supplementary Fig. 10
Supplementary Fig. 11
Supplementary Fig. 12

Supplementary Tables

Supplementary Table S1
Supplementary Table S2

Supplementary Fig. 1

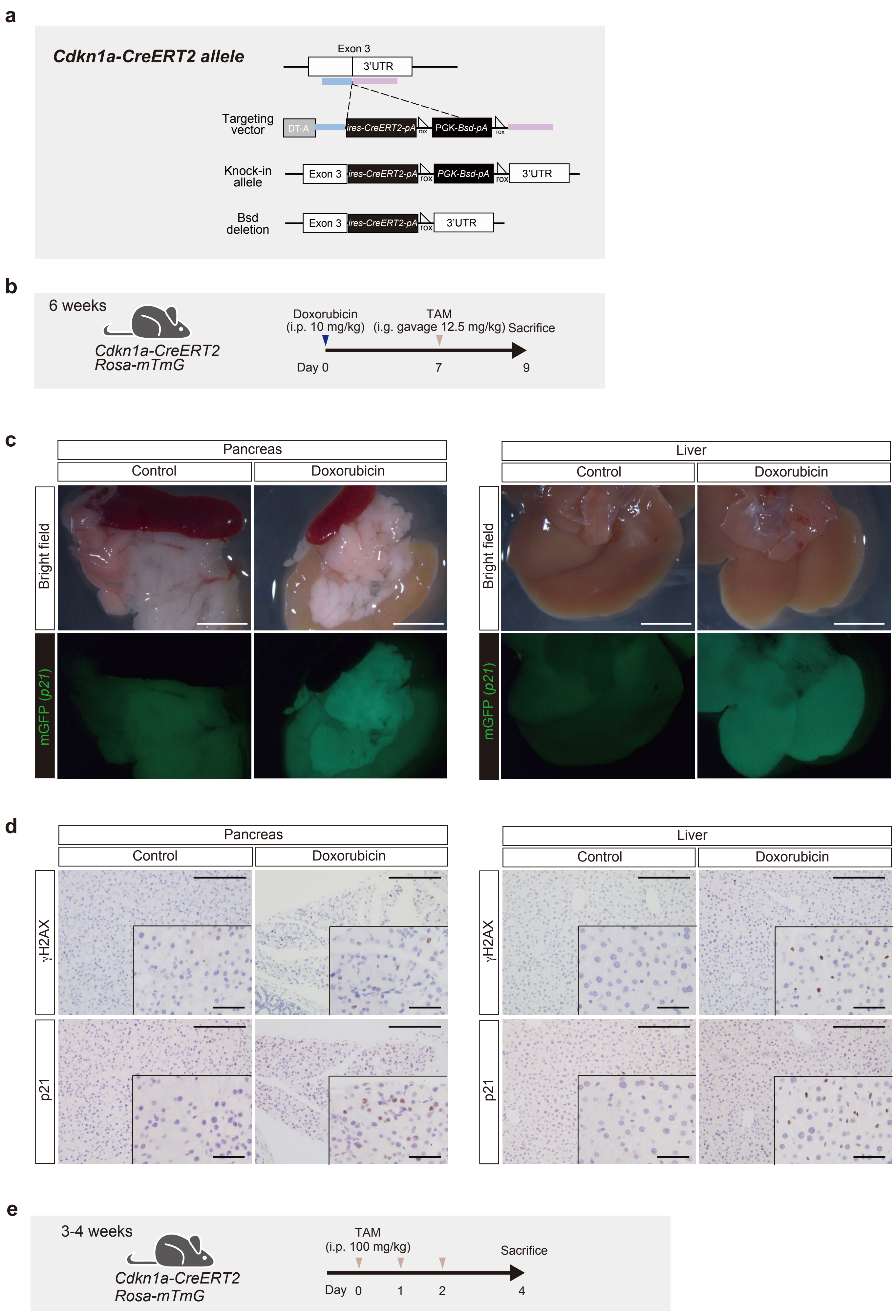

Supplementary Figure 1
Establishment of a *p21* reporter mouse model to visualize the DDR

a: Schematic representation of the genetic construct used to generate the *Cdkn1a* (*p21*)-*CreERT2* allele.

b: Experimental protocol for induction of DNA damage and visualization of sustained *p21* expression.

c: Macroscopic images showing mGFP expression in the pancreas and liver following DNA damage induction.

d: Immunohistochemical analysis of γH2AX and p21 expression. Doxorubicin treatment induces γH2AX and p21 expression.

e: Experimental protocol for visualization of *p21* expression in normal juvenile mice.

The doxorubicin-treated sample shown in b was obtained from a single mouse in one experiment, whereas the control samples were obtained from at least three mice. Scale bars: c, 5 mm; d, 200 µm; inset, 50 µm.

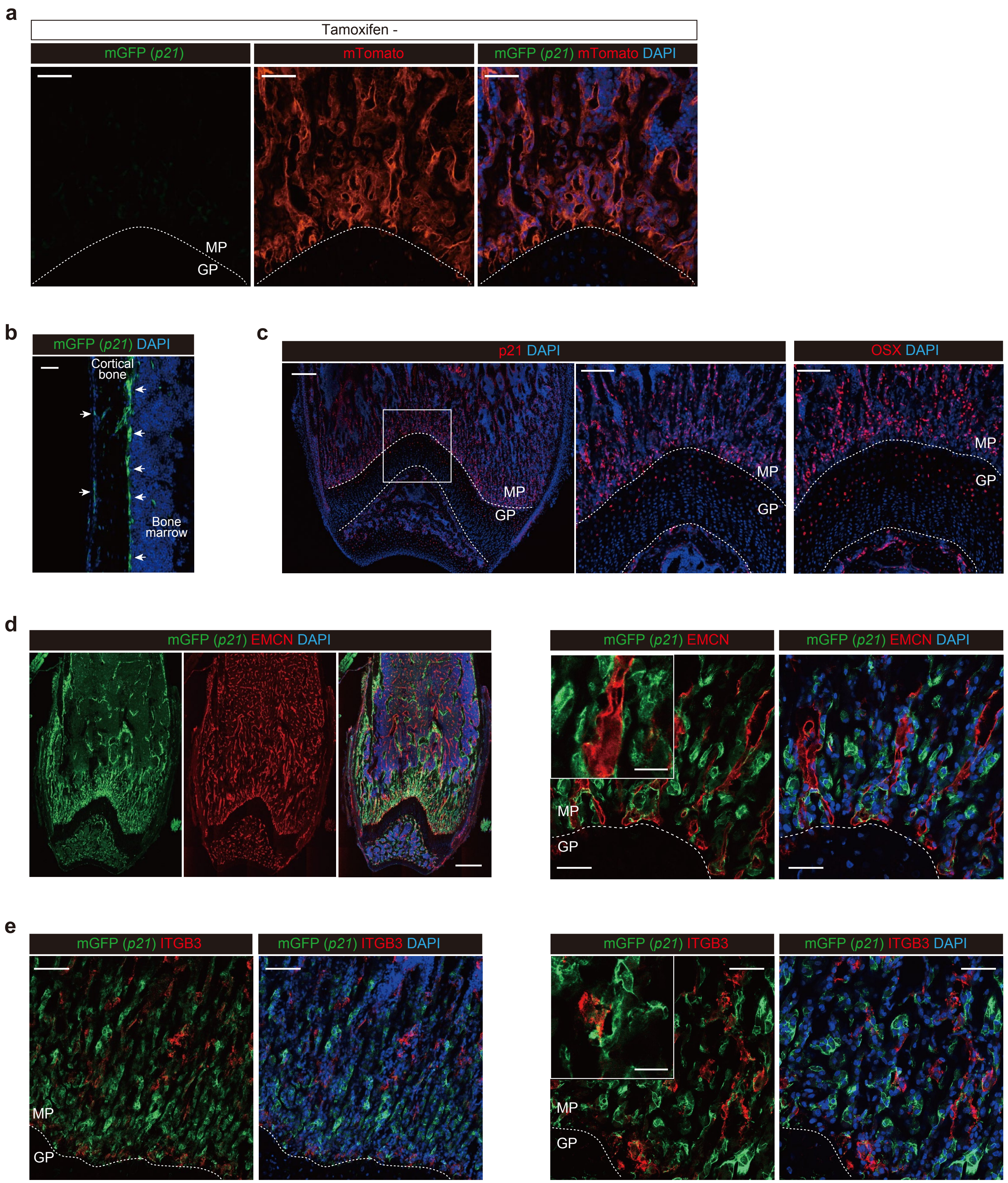
a
Tamoxifen -
mGFP (p21)
mTomato
mGFP (p21) mTomato DAPI
MP
GP
b
mGFP (p21) DAPI
Cortical bone
Bone marrow
c
p21 DAPI
OSX DAPI
MP
GP
d
mGFP (p21) EMCN DAPI
mGFP (p21) EMCN
mGFP (p21) EMCN DAPI
MP
GP
e
mGFP (p21) ITGB3
mGFP (p21) ITGB3 DAPI
MP
GP

Supplementary Figure 2
p21 expression in metaphyseal cells of juvenile mice

a: Absence of mGFP+ cells in the metaphysis without tamoxifen administration. *p21* reporter mice received corn oil injections following the same regimen as the tamoxifen-treated group. Only mTomato+ cells are detected in the control metaphysis.

b: *p21*-mGFP expression is detectable in cells located on the cortical bone surface.

c: Immunofluorescence analysis of p21 and OSX expression. p21 protein is predominantly expressed in metaphyseal cells, including frequent OSX+ osteoblasts.

d: Immunofluorescence analysis of EMCN, an endothelial cell marker, in mGFP+ cells. *p21*-mGFP+ cells do not express EMCN.

e: Immunofluorescence analysis of ITGB3, an osteoclast marker, in mGFP+ cells. *p21*-mGFP+ cells do not express ITGB3.

Representative images are from at least three biologically independent mice. Scale bars: a and b, 50 µm; c, left, 200 µm, middle and right, 100 µm; d, left, 500 µm, right, 50 µm, inset, 20 µm; e, left, 100 µm, e, right, 50 µm; e inset, 20 µm. MP, metaphysis; GP, growth plate.

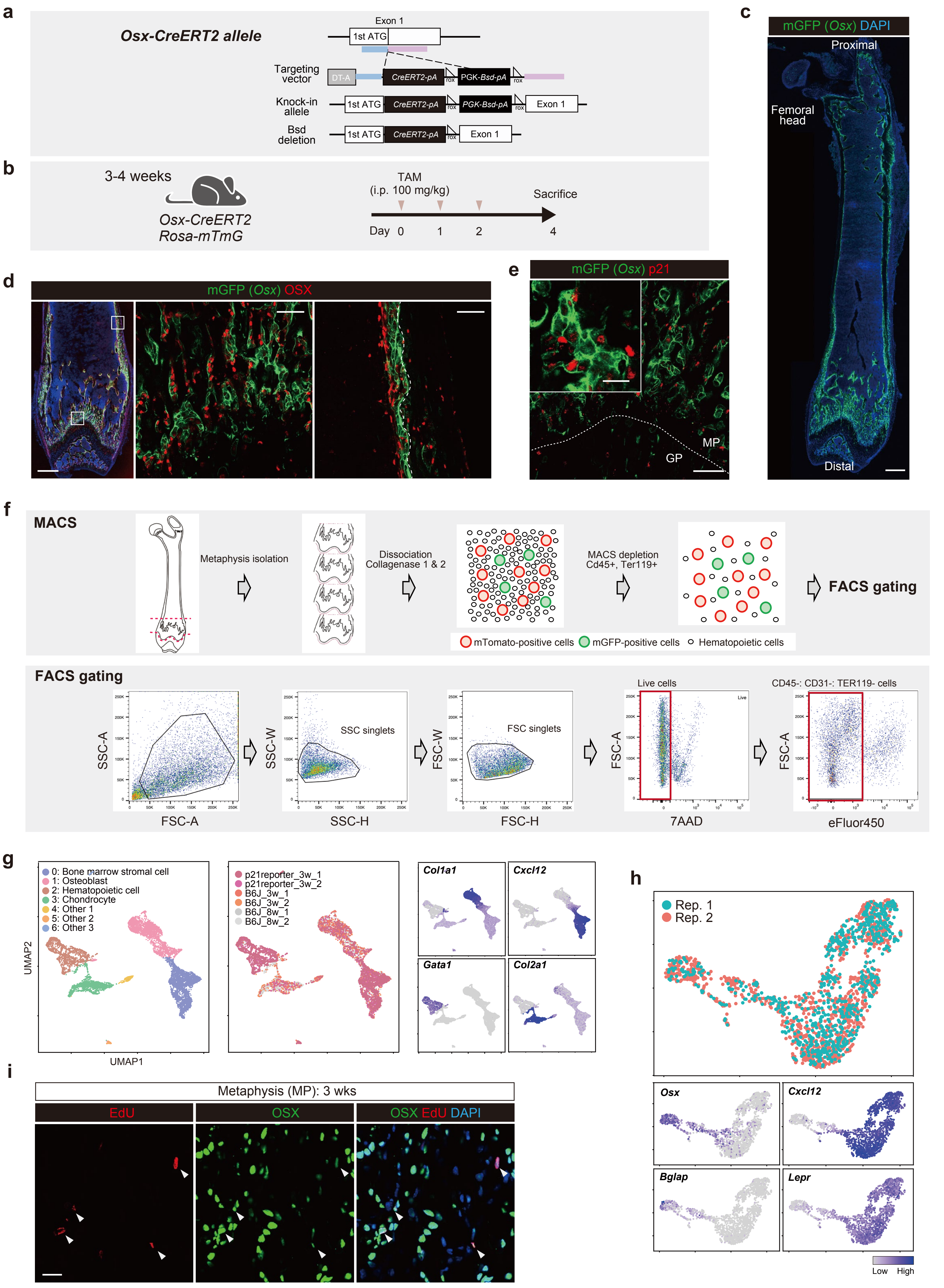
a
Osx-CreERT2 allele
Exon 1
1st ATG
Targeting vector
DT-A
CreERT2-pA
PGK-Bsd-pA
rox
Knock-in allele
Exon 1
Bsd deletion
b
3-4 weeks
Osx-CreERT2
Rosa-mTmG
TAM
(i.p. 100 mg/kg)
Sacrifice
Day 0 1 2 4
c
mGFP (Osx) DAPI
Proximal
Femoral head
Distal
d
mGFP (Osx) OSX
e
mGFP (Osx) p21
MP
GP
f
MACS
Metaphysis isolation
Dissociation
Collagenase 1 & 2
MACS depletion
Cd45+, Ter119+
FACS gating
mTomato-positive cells
mGFP-positive cells
Hematopoietic cells
FACS gating
SSC-A
FSC-A
SSC-W
SSC-H
SSC singlets
FSC-W
FSC-H
FSC singlets
Live cells
7AAD
CD45-: CD31-: TER119- cells
eFluor450
g
0: Bone marrow stromal cell
1: Osteoblast
2: Hematopoietic cell
3: Chondrocyte
4: Other 1
5: Other 2
6: Other 3
UMAP2
UMAP1
p21reporter_3w_1
p21reporter_3w_2
B6J_3w_1
B6J_3w_2
B6J_8w_1
B6J_8w_2
Col1a1
Cxcl12
Gata1
Col2a1
h
Rep. 1
Rep. 2
Osx
Cxcl12
Bglap
Lepr
Low High
i
Metaphysis (MP): 3 wks
EdU
OSX
OSX EdU DAPI

Supplementary Figure 3
Characterization of juvenile metaphyseal osteoblasts expressing *p21*

a: Schematic representation of the genetic construct used to generate the *Osx-CreERT2* allele.

b: Experimental protocol for visualization of *Osx* expression in normal juvenile mice.

c: mGFP expression in long bones of juvenile *Osx*-reporter mice.

d: Immunofluorescence analysis of OSX expression in the metaphysis of juvenile *Osx*-reporter mice. OSX is predominantly detected in *Osx*-mGFP+ cells in the metaphysis and on the cortical bone surface.

e: *Osx*-mGFP+ cells express p21 protein. MP, metaphysis; GP, growth plate.

f: Schematic overview of metaphyseal cell isolation and FACS gating strategy for all RNA-seq and scRNA-seq analyses in this study. Metaphyseal regions were isolated from long bones, enzymatically dissociated using collagenase I and II, and subjected to MACS depletion of CD45+ and Ter119+ cells before FACS analysis. Representative plots show sequential gating for intact cells, singlets, live cells (7AAD−), and CD45−CD31−Ter119− populations used for downstream analyses. This strategy applies to the FACS-based datasets shown in the main and supplementary figures.

g: Unsupervised clustering based on canonical marker gene expression identifies osteoblasts, chondrocytes, bone marrow stromal cells, and hematopoietic cells. Osteoblast and bone marrow stromal cell clusters were selected for further analyses.

h: Upper: UMAP visualization of scRNA-seq data from biological replicates. Bottom: Expression of canonical marker genes for metaphyseal osteoblasts and bone marrow stromal cells.

i: EdU-incorporated metaphyseal cells express OSX, an osteoblast marker.

Representative images are from at least three biologically independent mice. Scale bars: c and d (left), 500 µm; d (middle and right), e, and i, 50 µm; e (inset), 20 µm.

Supplementary Fig. 4

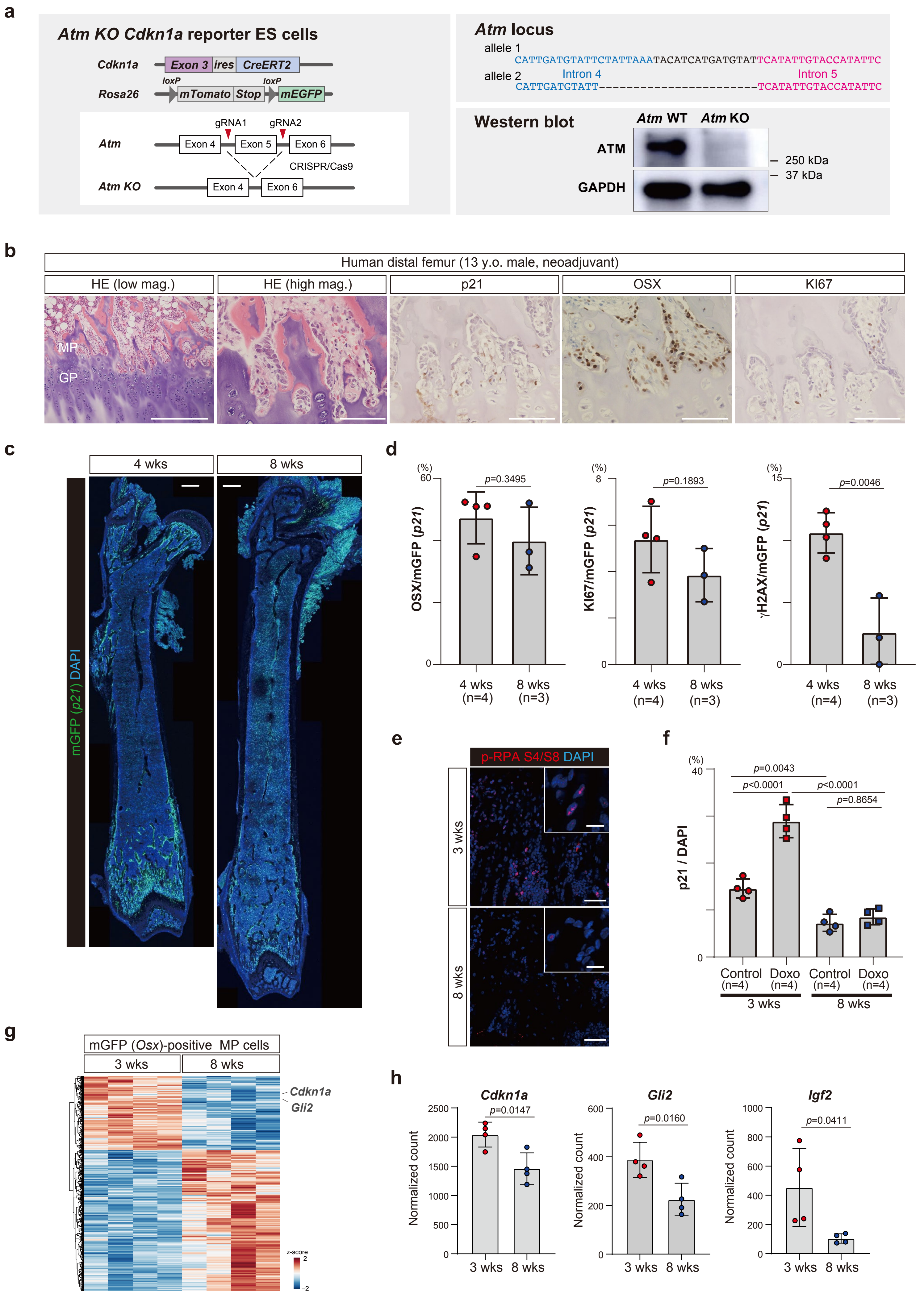

Supplementary Figure 4
Unique features of juvenile metaphyseal cells

a: Left: Schematic representation of genetic ablation of *Atm* in *p21*-reporter embryonic stem cells (*Atm* KO *p21*-reporter ESCs). Right: *Atm* disruption was confirmed by Sanger sequencing and western blot analysis. The western blot was performed once to confirm *Atm* disruption in the established *Atm* KO ESC clone used for chimera generation. Chimeric mice were generated using *Atm* KO ESCs.

b: Immunohistochemical analysis of human juvenile metaphysis. Juvenile metaphyseal cells frequently express OSX, p21, and KI67.

c: The number of *p21*-mGFP+ cells is markedly reduced in the metaphysis of adult mice. Tamoxifen was administered at 100 mg/kg in 3-week-old mice and 200 mg/kg in 8-week-old mice.

d: The proportion of KI67+ and γH2AX+ cells among *p21*-mGFP+ cells is significantly reduced in adult metaphysis.

e: Representative immunofluorescence images of p-RPA in juvenile and adult metaphysis.

f: Quantification of p21+ cells among metaphyseal cells following doxorubicin treatment in juvenile and adult mice. Doxorubicin markedly increases the frequency of p21+ metaphyseal osteoblasts in juvenile mice, whereas only modest effects are observed in adults.

g: Heatmap of RNA-seq analysis of osteoblasts from juvenile and adult mice. Gene expression levels are shown as z-scores.

h: Normalized RNA-seq counts of *Cdkn1a*, *Gli2*, and *Igf2*. Each dot represents an RNA-seq sample generated from pooled *Osx*+ osteoblasts. Each cohort consisted of three biological replicates.

Source data are provided as a Source Data file. Data are shown as mean ± SD. For all relevant panels, n indicates the number of mice analyzed. Representative images are from at least three biologically independent mice. For comparisons between two groups, statistical significance was determined by two-sided unpaired Student's t-test. For multiple pairwise comparisons among more than two groups, statistical significance was determined by two-sided one-way ANOVA with Tukey's adjustment for multiple comparisons. Exact P values are indicated in the figure. Scale bars: b, HE left, 500 µm, HE right and immunohistochemistry, 100 µm; c, 500 µm; e, 50 µm; inset, 12.5 µm.

Supplementary Fig. 5

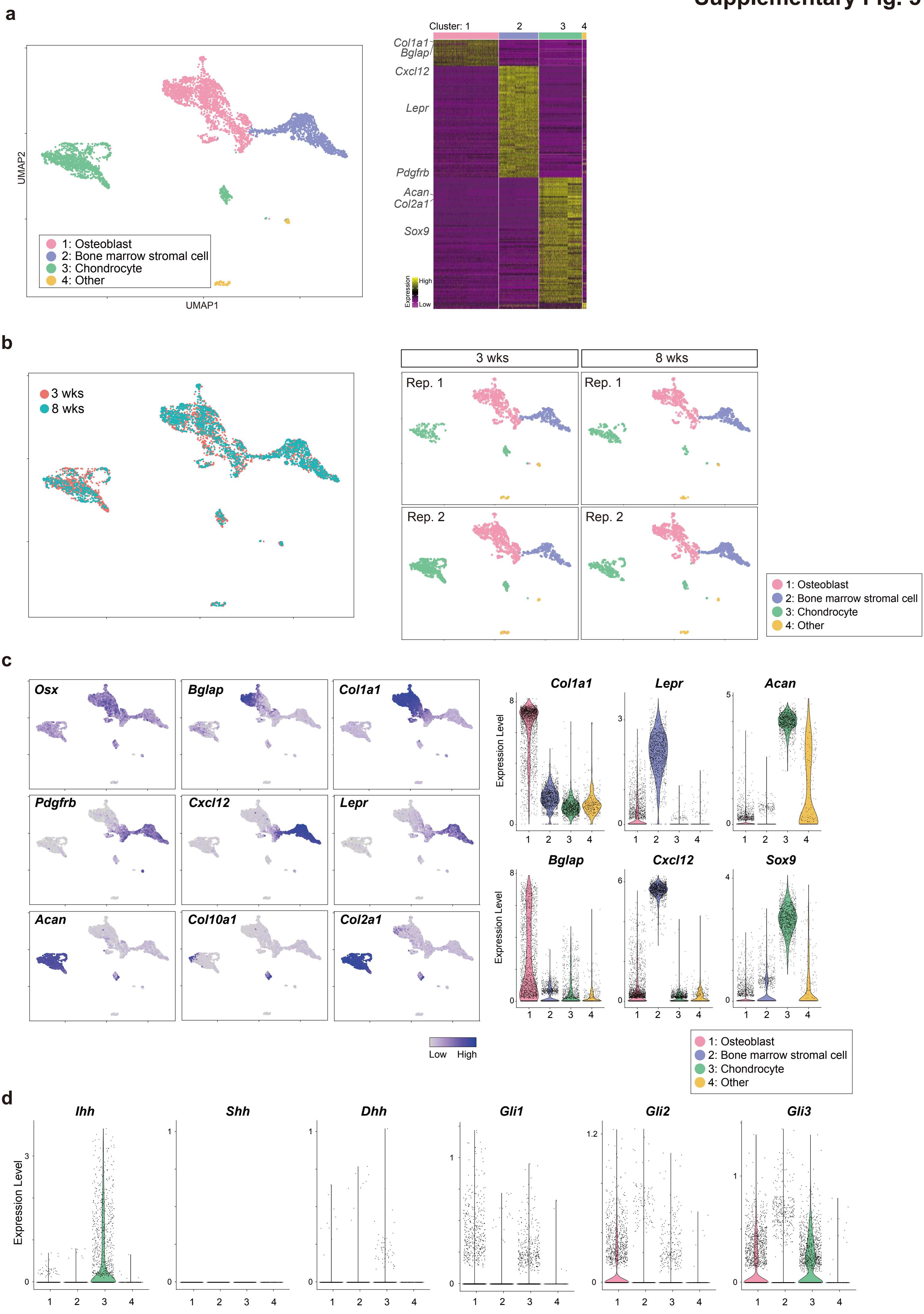

Supplementary Figure 5
scRNA-seq analysis of juvenile and adult metaphyseal cells

a: Unsupervised clustering based on canonical marker gene expression identifies osteoblasts, chondrocytes, and bone marrow stromal cells.

b: Left: UMAP visualization of scRNA-seq data from juvenile and adult metaphyseal cells. Right: UMAP plots for individual biological samples.

c: Expression patterns of lineage marker genes shown in UMAP plots (left) and corresponding cluster-wise expression levels (right).

d: Expression of Hedgehog ligands and downstream transcription factors.

Supplementary Fig. 6

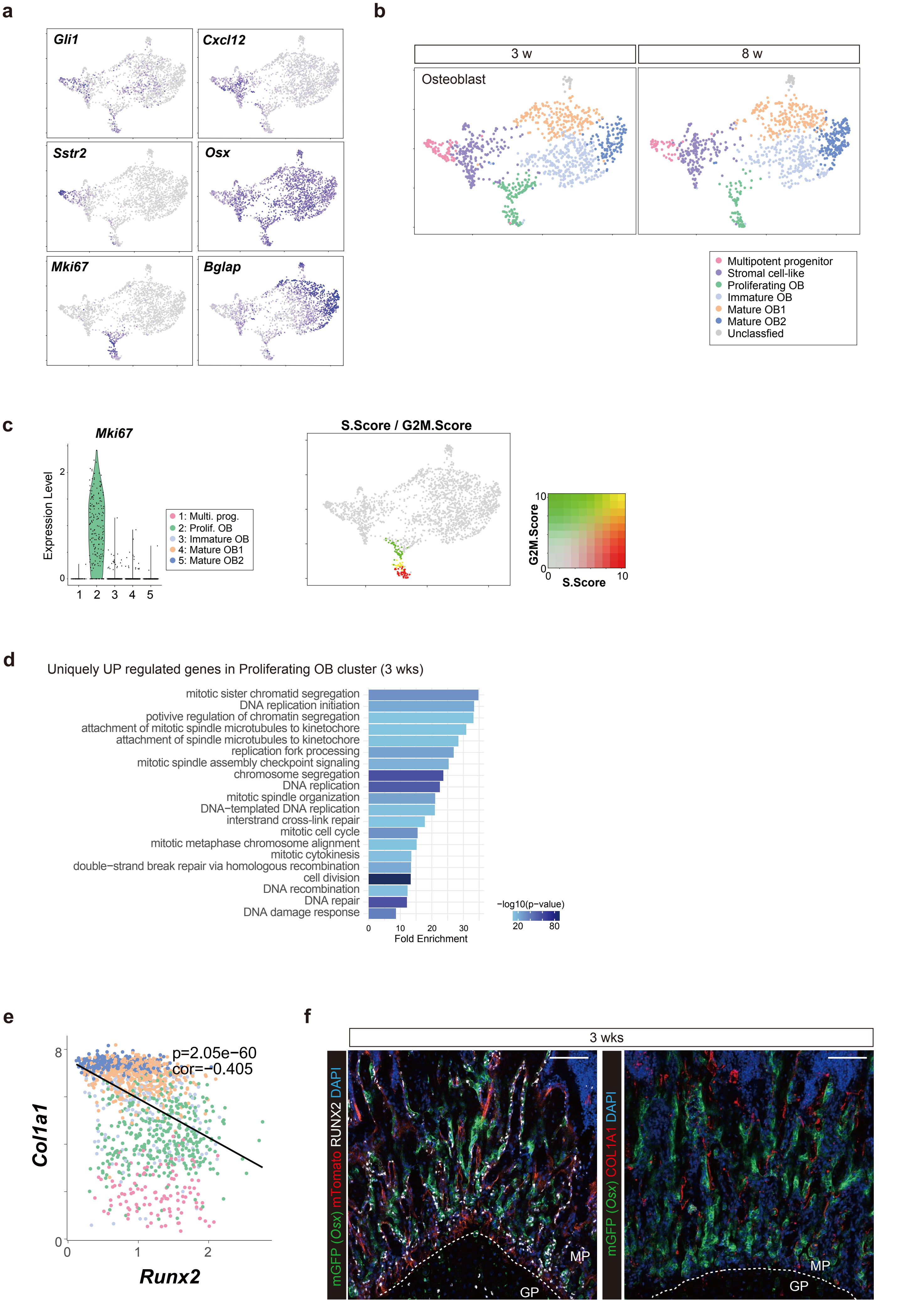

Supplementary Figure 6
scRNA-seq analysis of juvenile and adult osteoblast-lineage cells

a: UMAP plots showing expression patterns of marker genes in osteoblast-lineage cells.

b: Subclustering of juvenile and adult osteoblast-lineage cells. Clusters were annotated based on lineage marker gene expression profiles.

c: Left: Expression levels of *Mki67* in each cluster. Right: Cell cycle scores visualized in UMAP plots. Active proliferation is restricted to the proliferating osteoblast (Proliferating OB) cluster.

d: Gene Ontology analysis of genes uniquely upregulated in the juvenile Proliferating OB cluster (adjusted $p < 0.05$, log2 fold change > 1), showing enrichment of pathways related to DNA replication and DDR.

e: Single-cell analysis showing negative correlation between *Runx2* and *Col1a1* expression. Pearson's correlation coefficients and P values were calculated using the cor.test function in R (version 4.3.2). All tests were two-sided with the null hypothesis of no correlation ($\rho = 0$).

f: Immunofluorescence analysis of RUNX2 and COL1A1 in the metaphysis of *Osx*-reporter mice. RUNX2 expression decreases, whereas COL1A1 expression increases with increasing distance from the growth plate. MP, metaphysis; GP, growth plate. Scale bar: f, 50 μm.

Representative images are from at least three biologically independent mice.

Supplementary Fig. 7

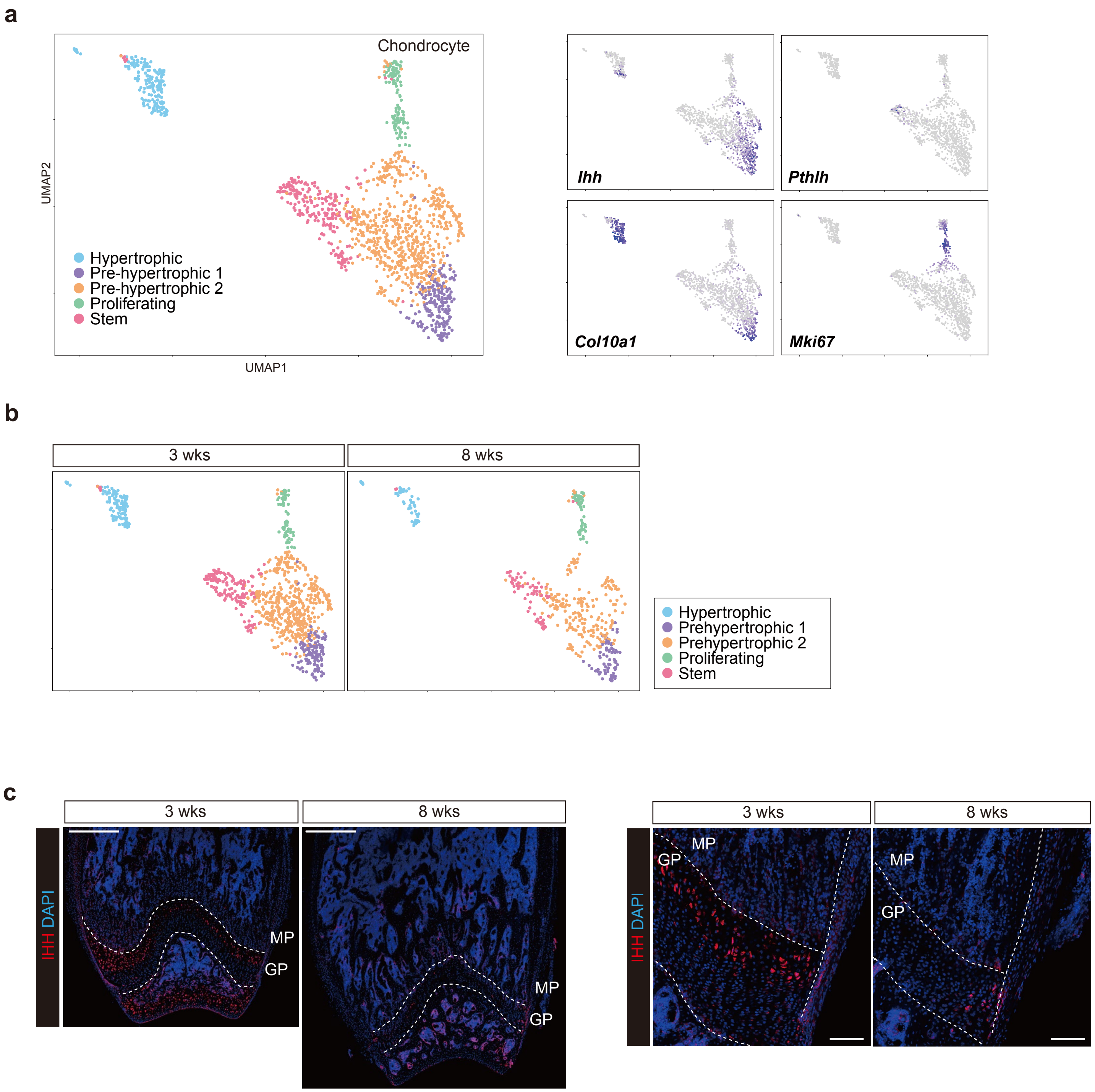

Supplementary Figure 7
scRNA-seq analysis of juvenile and adult chondrocyte-lineage cells

a: Left: Subclustering of chondrocyte-lineage cells. Right: UMAP plots showing expression patterns of marker genes in chondrocyte-lineage cells.

b: Subclustering of juvenile and adult chondrocyte-lineage cells.

c: Immunofluorescence analysis of IHH expression in juvenile and adult metaphysis. In juvenile mice, IHH is predominantly expressed in prehypertrophic chondrocytes within the growth plate, whereas in adult mice its expression is largely restricted to the perichondrium. MP, metaphysis; GP, growth plate.

Representative images are from at least three biologically independent mice. Scale bars: c, left, 500 μm; right, 50 μm.

Supplementary Fig. 8

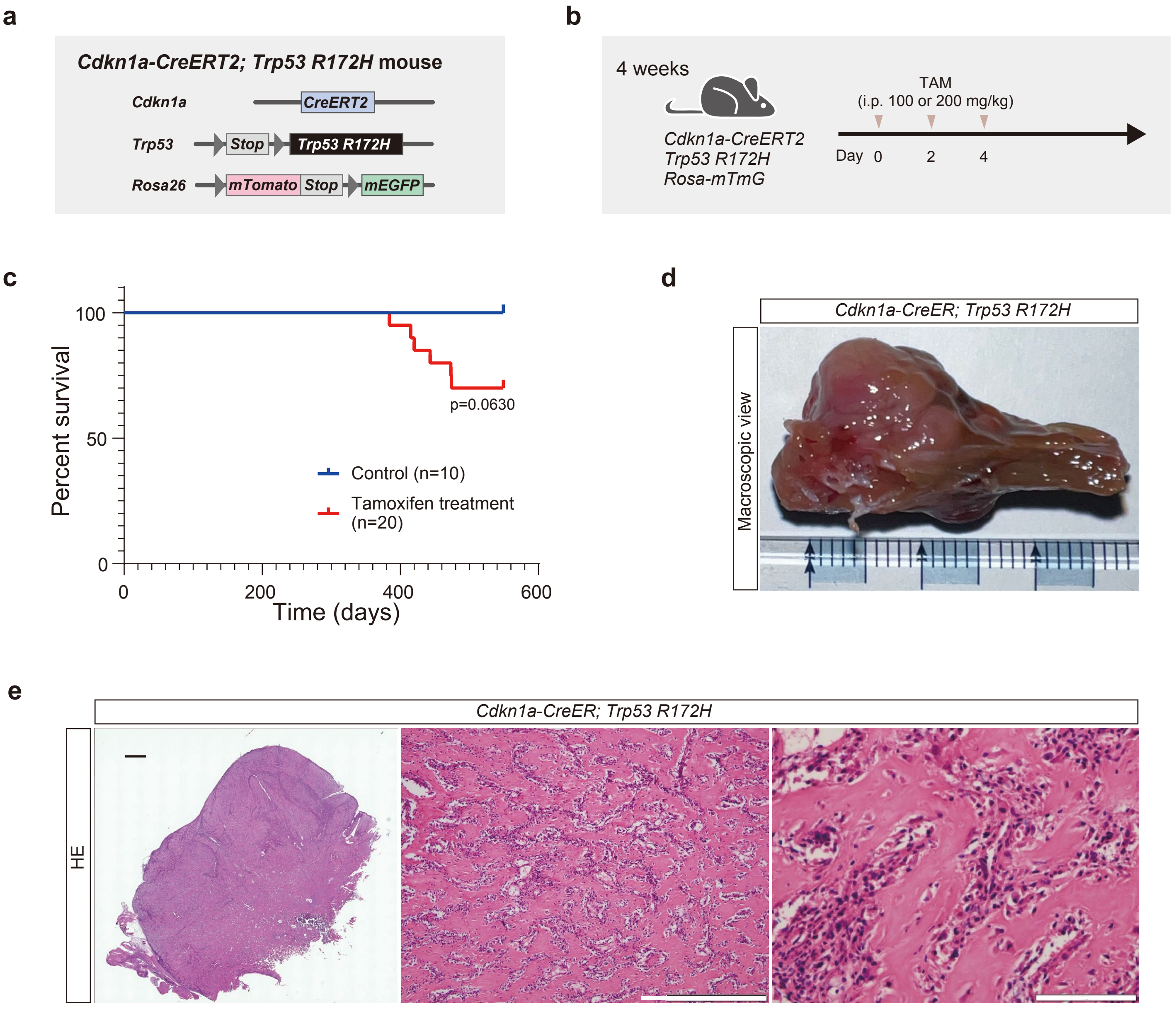

Supplementary Figure 8
p53 inactivation in *p21*+ cells induces osteosarcoma development

a: Schematic representation of the genetic construct used to induce p53 inactivation in *p21*+ cells *in vivo*.

b: Experimental protocol for induction of p53 inactivation in juvenile mice.

c: Survival curve of mice with p53 inactivation in *p21*+ cells. Statistical significance was assessed using the log-rank (Mantel–Cox) test. n indicates the number of mice analyzed.

d: Macroscopic image showing spinal tumors in *p53*-mutant mice.

e: Histological analysis of a representative tumor from *p53*-mutant mice. The tumor exhibits features of osteogenic osteosarcoma, including extracellular osteoid formation. Similar histological features were confirmed in two independent tumors.

Scale bars: e, left, 1,000 µm; middle, 500 µm; right, 100 µm.

Supplementary Fig. 9

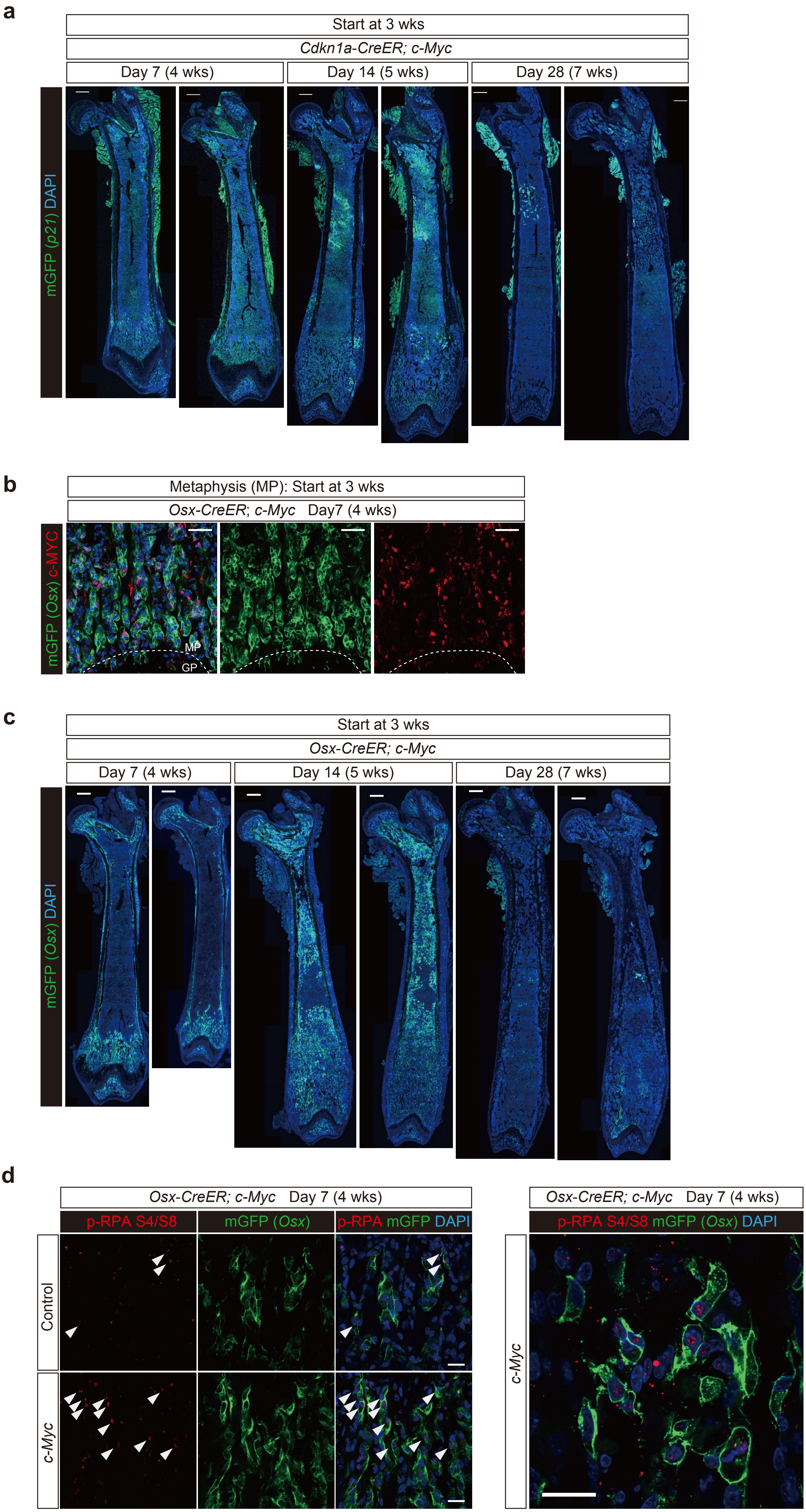

Supplementary Figure 9
Forced expression of *c-Myc* induces transient proliferation of juvenile osteoblasts

a: Transient expansion of mGFP+ cells following *c-Myc* transduction in the *Cdkn1a-CreERT2* model. Expanded *p21*-mGFP+ cells decline by day 28 (7 weeks of age). The day 14 and day 28 femurs shown are identical to those shown in Fig. 5i.

b: Immunofluorescence analysis of c-MYC expression in the metaphysis of *c-Myc*-induced mice. c-MYC is detected in *Osx*-mGFP+ osteoblasts.

c: Transient expansion of osteoblasts following *c-Myc* transduction in the *Osx-CreERT2* model. Expanded mGFP+ osteoblasts decline by day 28 (7 weeks of age). The day 7 femur shown in the left panel is identical to that shown in Fig. 5h.

d: Representative images of p-RPA staining in the femoral metaphysis following *c-Myc* induction in the *Osx-CreERT2* model.

Representative images are from at least three biologically independent mice. Scale bars: a and c, 500 μm; b, 50 μm; d, 20 μm.

Supplementary Fig. 10

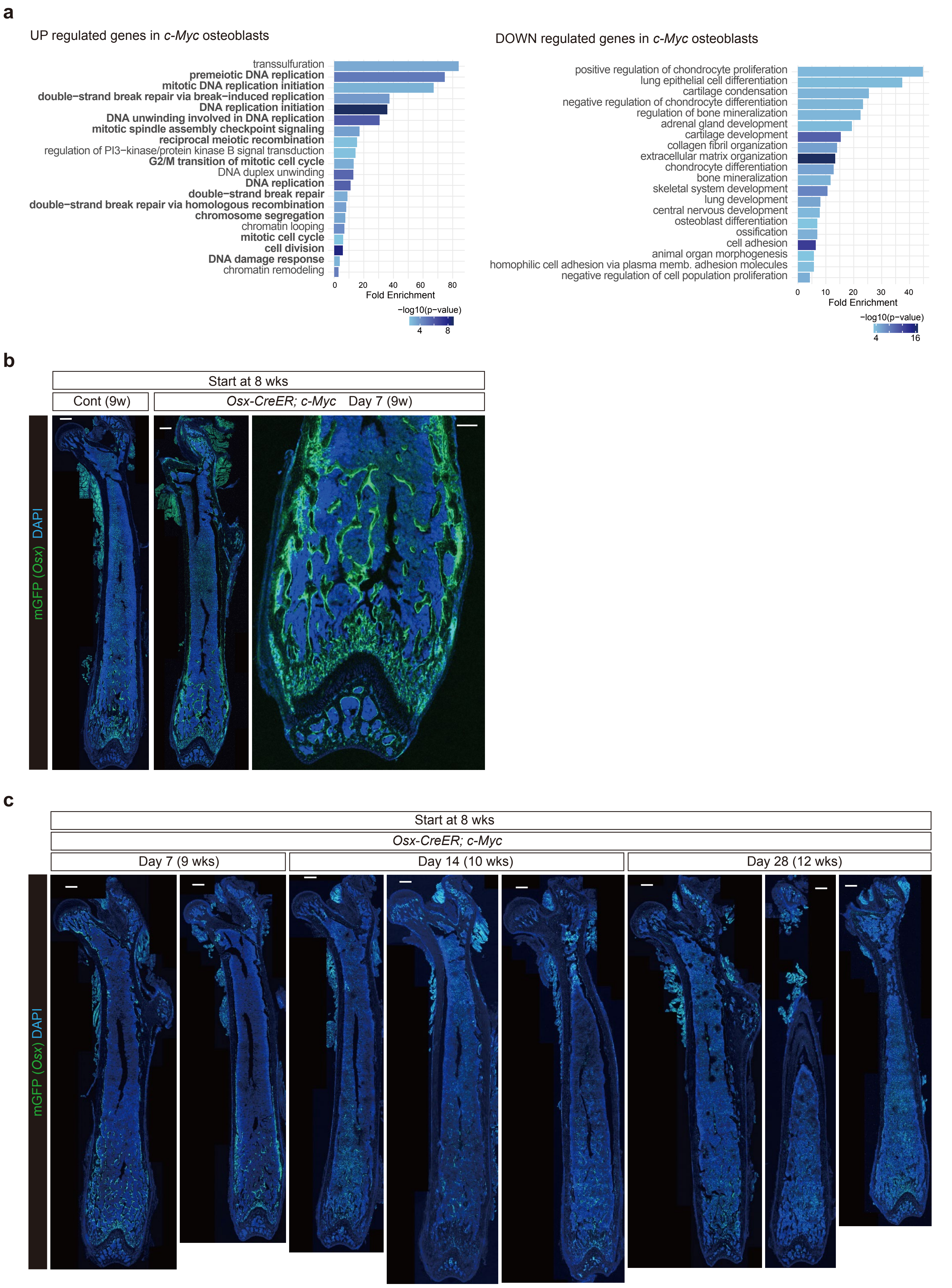

Supplementary Figure 10
Forced expression of *c-Myc* induces proliferation of juvenile but not adult osteoblasts

a: Gene Ontology analysis of genes upregulated following *c-Myc* transduction reveals enrichment of pathways related to DNA replication and DDR. Bold terms indicate gene ontology terms related to DNA replication or DDR. Differentially expressed genes were identified based on TPM values, with upregulated genes defined as adjusted $p < 0.05$ and log2 fold change > 3, and downregulated genes as adjusted $p < 0.05$ and log2 fold change < –3.

b: Effects of *c-Myc* transduction in adult osteoblasts. In contrast to juvenile mice, *c-Myc* expression does not induce robust osteoblast expansion in adult mice.

c: Time-course analysis of femurs following *c-Myc* transduction in adult *Osx*+ osteoblasts. *c-Myc* expression does not induce expansion of adult *Osx*+ osteoblasts.

Representative images are from at least three biologically independent mice. Scale bars: b, left and middle, 500 μm, right, 200 μm; c, 500 μm.

Supplementary Fig. 11

a

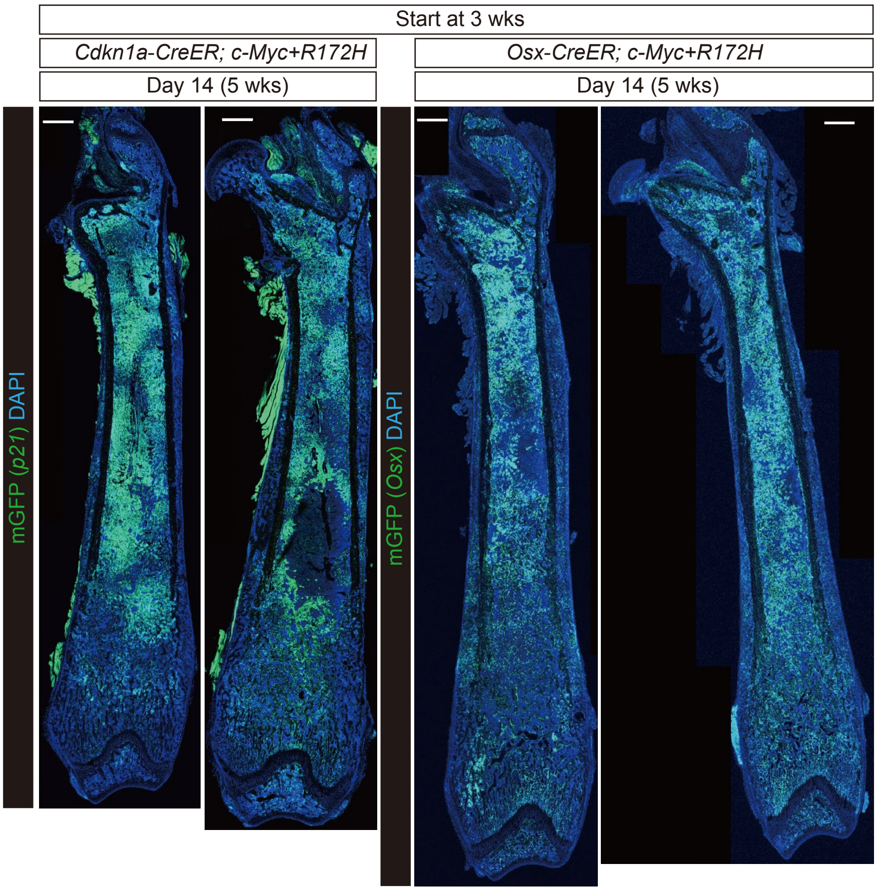

b

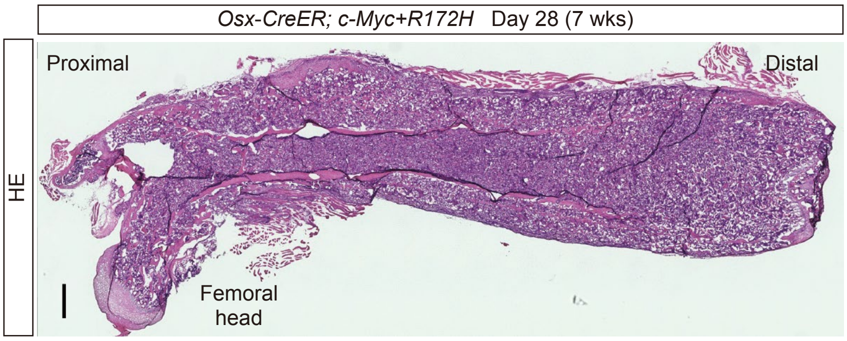

c

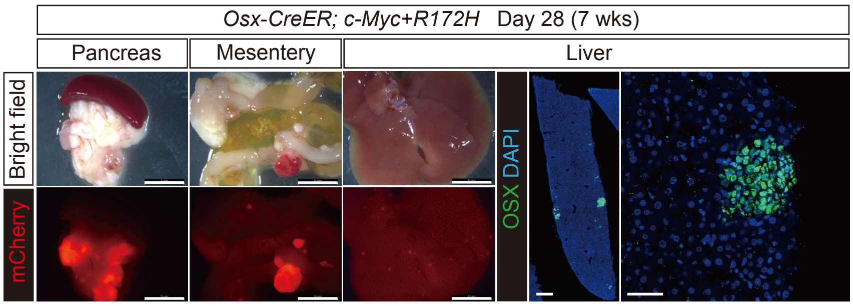

Supplementary Figure 11
p53 inactivation enables continuous expansion of *c-Myc*-induced juvenile osteoblasts

a: Expansion of mGFP+ cells following combined induction of *c-Myc* and *Trp53R172H* in the *Cdkn1a-CreERT2* and *Osx-CreERT2* models. mGFP+ cells expand with similar kinetics in both models.

b: HE staining of femurs from *c-Myc*; *Trp53R172H* mice in the *Osx-CreERT2* model. Mice were analyzed at 7 weeks of age, 28 days after the initial tamoxifen treatment. The entire femur is replaced by cancerous tissue exhibiting histological features characteristic of osteogenic osteosarcoma.

c: Multiple metastases derived from *Osx*+ osteoblasts. OSX+ cells are disseminated in the pancreas, mesentery, and liver 28 days after the combined induction of p53 inactivation and *c-Myc* expression.

Representative images are from at least three biologically independent mice. Scale bars: a and b, 500 µm; c, stereomicroscopic images, 5 mm, immunofluorescence images, left, 200 µm, right, 50 µm.

Supplementary Fig. 12

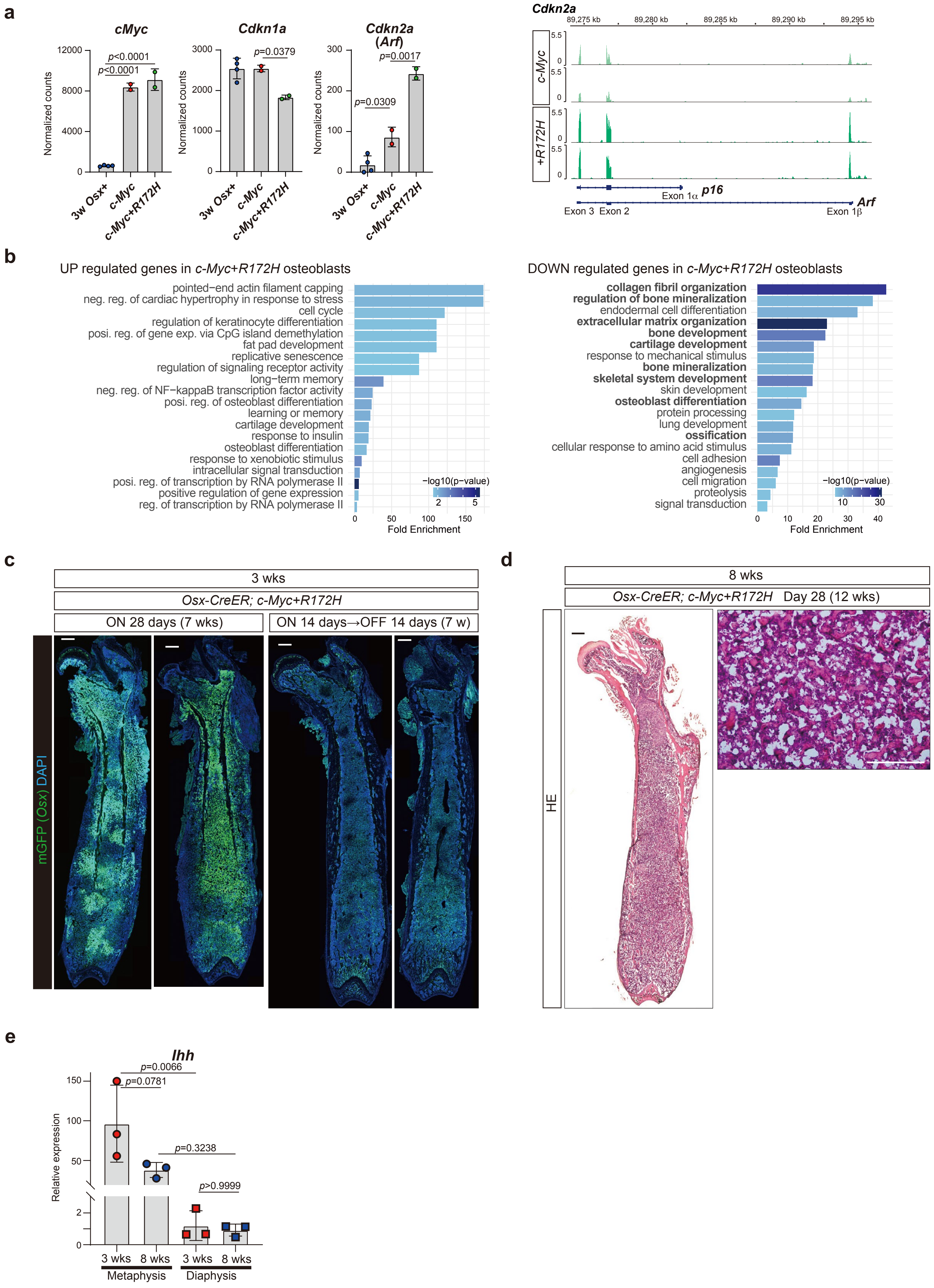

Supplementary Figure 12
p53 inactivation enables expansion of *c-Myc*-induced osteoblasts even in adult mice

a: Normalized RNA-seq counts of *c-Myc*, *Cdkn1a*, and *Cdkn2a* in *Osx*+ osteoblasts. Each dot represents an RNA-seq sample generated from pooled *Osx*+ osteoblasts. Sample sizes were n = 4 for 3w *Osx*+ and n = 2 for *c-Myc* and *c-Myc+R172H*. Right, Integrative Genomics Viewer (IGV) tracks showing that *Arf* expression, elevated in *c-Myc*-induced osteoblasts, is further increased upon p53 inactivation, whereas *p16* expression is undetectable.

b: Gene Ontology analysis of genes downregulated following p53 inactivation reveals suppression of osteoblast differentiation. Bold terms indicate gene ontology terms related to osteoblast differentiation, skeletal development, or extracellular matrix organization. Differentially expressed genes were identified based on TPM values with adjusted $p < 0.05$.

c: Withdrawal of *c-Myc* expression suppresses abnormal growth of *c-Myc+Trp53R172H* osteoblasts. *c-Myc* was induced in p53-inactivated osteoblasts for 2 weeks starting at 3 weeks of age, followed by a 2-week withdrawal period with tetracycline administration in drinking water. Expansion of mGFP+ osteoblasts is no longer observed, indicating that sustained *c-Myc* expression is required for abnormal growth.

d: Combined induction of *c-Myc* and p53 inactivation in adult osteoblasts (8 weeks of age) results in osteosarcoma formation. HE staining shows extensive replacement of femoral tissue by osteogenic osteosarcoma.

e: RT–qPCR analysis of *Ihh* expression in metaphyseal and diaphyseal regions isolated from juvenile and adult mice. Each dot represents one biologically independent mouse. Each cohort consisted of three biological replicates.

Source data are provided as a Source Data file. Data are shown as mean ± SD. Representative images are from at least three biologically independent mice. Statistical significance was determined by two-sided one-way ANOVA with Tukey's adjustment for multiple comparisons. Exact P values are indicated in the figure. Scale bars: c, 500 µm; d, left, 500 µm, right, 200 µm.

Table S1: Gene sets significantly enriched in c-Myc+Trp53 R172H osteoblasts compared to c-Myc osteoblasts

ID	setSize	enrichmentScore	NES	pvalue	p.adjust	qvalue
HALLMARK_EPITHELIAL_MESENCHYMAL_TRANSITION	117	-0.752833723	-2.9232197	1.00E-10	2.45E-09	1.37E-09
HALLMARK_MYOGENESIS	83	-0.688715142	-2.5250784	1.00E-10	2.45E-09	1.37E-09
HALLMARK_ANGIOGENESIS	21	-0.823266463	-2.2828876	1.30E-07	2.13E-06	1.19E-06
HALLMARK_COAGULATION	46	-0.680676648	-2.2436315	2.39E-07	2.93E-06	1.64E-06
HALLMARK_E2F_TARGETS	184	0.400193533	1.92666344	3.51E-07	3.44E-06	1.92E-06
HALLMARK_MTORC1_SIGNALING	172	-0.463569883	-1.8893604	3.86E-06	3.15E-05	1.76E-05
HALLMARK_P53_PATHWAY	137	-0.467815761	-1.8484099	2.10E-05	1.47E-04	8.23E-05
HALLMARK_GLYCOLYSIS	132	-0.475046387	-1.8698716	5.10E-05	3.12E-04	1.74E-04
HALLMARK_HYPOXIA	122	-0.457379539	-1.7813316	8.67E-05	4.25E-04	2.37E-04
HALLMARK_OXIDATIVE_PHOSPHORYLATION	184	0.348926078	1.67984503	8.41E-05	4.25E-04	2.37E-04
HALLMARK_APOPTOSIS	98	-0.467889979	-1.7639665	3.92E-04	0.001599086	8.93E-04
HALLMARK_G2M_CHECKPOINT	179	0.333565561	1.59846451	3.67E-04	0.001599086	8.93E-04
HALLMARK_APICAL_JUNCTION	92	-0.478646978	-1.7863759	4.67E-04	0.001760041	9.83E-04
HALLMARK_KRAS_SIGNALING_UP	65	-0.512462535	-1.814398	9.73E-04	0.003233225	0.001805883
HALLMARK_TNFA_SIGNALING_VIA_NFKB	105	0.374399174	1.64525856	9.90E-04	0.003233225	0.001805883
HALLMARK_INTERFERON_GAMMA_RESPONSE	77	-0.467304788	-1.6863765	0.002622559	0.008031585	0.004485955
HALLMARK_UV_RESPONSE_DN	94	-0.429517685	-1.6077497	0.004564916	0.0131577	0.007349091
HALLMARK_UNFOLDED_PROTEIN_RESPONSE	104	-0.415853924	-1.5843632	0.005729795	0.015597774	0.008711968
HALLMARK_INFLAMMATORY_RESPONSE	53	-0.491349575	-1.6845264	0.008127451	0.019912255	0.011121775
HALLMARK_COMPLEMENT	84	-0.428239188	-1.5741866	0.007865954	0.019912255	0.011121775
HALLMARK_PROTEIN_SECRETION	74	-0.432706995	-1.5564727	0.009248462	0.021579745	0.012053134
HALLMARK_IL2_STAT5_SIGNALING	101	-0.389774093	-1.4746551	0.016198877	0.036079317	0.020151713
HALLMARK_ANDROGEN_RESPONSE	72	-0.409976972	-1.4668871	0.019970198	0.042545205	0.023763165

Supplementary Table S1

Gene sets significantly enriched in *c-Myc+Trp53 R172H* osteoblasts compared with *c-Myc* osteoblasts

Gene set enrichment analysis was performed using MSigDB Hallmark gene sets. Genes were ranked by log2 fold change between *c-Myc+Trp53 R172H* and *c-Myc* osteoblasts. Nominal P values were calculated by permutation-based GSEA, and P values were adjusted for multiple comparisons using the Benjamini–Hochberg method. Positive and negative normalized enrichment scores indicate enrichment in *c-Myc+Trp53 R172H* and *c-Myc* osteoblasts, respectively.

Table S2: Primers

Name	Sequence
Genotyping	
Osx_CreERT2_ Fw	CTTGAGAGGAGACGGGACAG
Osx_CreERT2_Rv	TCTTGCGAACCTCATCACTC
Osx_CreERT2_Rv for WT	TCTGACCCCTGGCTATGTTC
p21_ires_CreERT2_Fw	TAGAGCCCTGTCCCTCTGAC
p21_ires_CreERT2_Rv	AGACCCCTAGGAATGCTCGT
p21_ires_CreERT2_Rv for WT	GTTTGGAGACTGGGAGAGGG
Rosa26_mTmG_Fw	GTAAGGGAGCTGCAGTGGAG
Rosa26_mTmG_Rv	GGGGCGTACTTGGCATATGA
Rosa26_mTmG_Rv for WT	ACCTGTTCAATTCCCCTGCA
Rosa26_LSL_tTA_Fw	AAAGTCGCTCTGAGTTGTTAT
Rosa26_LSL_tTA_Rv	GCGAAGAGTTTGTCCTCAACC
Rosa26_LSL_tTA_Rv for WT	GGAGCGGGAGAAATGGATATG
Trp53_LSL_R172H_Fw	AGGTGTGGCTTCTGGCTTC
Trp53_LSL_R172H_Rv	CCAGCTCATTCCTCCCACTC
Trp53_LSL_R172H_Rv for WT	GAAACTTTTCACAAGAACCAGATCA
Col1a1_TetO_cMyc_mCherry_Fw	TTCACCAGTTCAATCATCCCAGGTGC
Col1a1::TetO-cMyc_mCherry_RV	GCAGAAGCGCGGCCGTCTGG
Col1a1::TetO-cMyc_mCherry_RV for WT	CAGCTACCCCTCCATGTGTGAC
RT-qPCR	
Ihh-Fw	CCCAACTACAATCCCGACAT
Ihh-Rv	AGTTCAGACGGTCCTTGCAG
Gapdh-Fw	AGGTCGGTGTGAACGGATTTG
Gapdh-Rv	TGTAGACCATGTAGTTGAGGTCA

Supplementary Table S2
Primers used in this study
