# Supplementary material for: Inherent tissue homeostasis of the juvenile metaphysis provides a foundation for osteosarcoma development
Source: Nat Commun. 2026 Jun 26;17:6241. doi: 10.1038/s41467-026-74929-6 (PMC13373234; doi:10.1038/s41467-026-74929-6)
Supplement: Supplementary file 2 — Reporting Summary [file 41467_2026_74929_MOESM2_ESM.pdf]

nature portfolio

Corresponding author(s): Yasuhiro Yamada

Last updated by author(s): 2026/6/8

Reporting Summary

Nature Portfolio wishes to improve the reproducibility of the work that we publish. This form provides structure for consistency and transparency in reporting. For further information on Nature Portfolio policies, see our Editorial Policies and the Editorial Policy Checklist.

Statistics

For all statistical analyses, confirm that the following items are present in the figure legend, table legend, main text, or Methods section.

n/a	Confirmed	
☐	☒	The exact sample size (*n*) for each experimental group/condition, given as a discrete number and unit of measurement
☐	☒	A statement on whether measurements were taken from distinct samples or whether the same sample was measured repeatedly
☐	☒	The statistical test(s) used AND whether they are one- or two-sided<br>*Only common tests should be described solely by name; describe more complex techniques in the Methods section.*
☒	☐	A description of all covariates tested
☐	☒	A description of any assumptions or corrections, such as tests of normality and adjustment for multiple comparisons
☐	☒	A full description of the statistical parameters including central tendency (e.g. means) or other basic estimates (e.g. regression coefficient) AND variation (e.g. standard deviation) or associated estimates of uncertainty (e.g. confidence intervals)
☐	☒	For null hypothesis testing, the test statistic (e.g. *F*, *t*, *r*) with confidence intervals, effect sizes, degrees of freedom and *P* value noted<br>*Give P values as exact values whenever suitable.*
☒	☐	For Bayesian analysis, information on the choice of priors and Markov chain Monte Carlo settings
☒	☐	For hierarchical and complex designs, identification of the appropriate level for tests and full reporting of outcomes
☒	☐	Estimates of effect sizes (e.g. Cohen's *d*, Pearson's *r*), indicating how they were calculated

Our web collection on statistics for biologists contains articles on many of the points above.

Software and code

Policy information about availability of computer code

Data collection	Imaging: IX71 (OLYMPUS), LSM900 (Zeiss), BZ-X710 (KEYENCE)<br>FACS: FACS Melody (BD)

Data analysis

Microsoft Excel for Mac (16.97.2)
GraphPad Prism 8 (v8.4.3)
Flowjo (v10.10.0)
Image J 64bit (v1.54)
Fiji (v1.54)
Cell Ranger pipelines (v7.2.0)
Seurat package (v5.0.1)
Doublet Finder (v2.0.4)
Python package scVelo (v0.3.0. 2)
Velocyto (v0.17.17)
cutadapt (v4.6)
STAR (v2.7.11a)
HTSeq-count (v2.0.5)
DESeq2 (v1.42.0)
HOMER (v4.11)
DAVID (v6.8)
R (v4.3.2)
Adobe Illustrator (v30.5.1)

For manuscripts utilizing custom algorithms or software that are central to the research but not yet described in published literature, software must be made available to editors and reviewers. We strongly encourage code deposition in a community repository (e.g. GitHub). See the Nature Portfolio guidelines for submitting code & software for further information.

Data

Policy information about availability of data

All manuscripts must include a data availability statement. This statement should provide the following information, where applicable:

- Accession codes, unique identifiers, or web links for publicly available datasets
- A description of any restrictions on data availability
- For clinical datasets or third party data, please ensure that the statement adheres to our policy

RNA-seq and scRNA-seq data have been deposited in the Gene Expression Omnibus under accession codes GSE297565 and GSE333438.

Research involving human participants, their data, or biological material

Policy information about studies with human participants or human data. See also policy information about sex, gender (identity/presentation), and sexual orientation and race, ethnicity and racism.

Reporting on sex and gender

Sex was reported for both animal experiments and human samples where available. Male mice were used for RNA-seq and scRNA-seq experiments, whereas both male and female mice were used for other experiments. Sex was not considered as a biological variable in the study design or statistical analyses because the study focused on age- and tissue context-dependent osteoblast responses. For human samples, sex was determined from clinical records, and sex/gender-based comparisons were not performed because the samples were used only to confirm p21 expression in juvenile metaphyseal osteoblasts by immunohistochemistry.

Reporting on race, ethnicity, or other socially relevant groupings

Not applicable. Human tissue samples were used only to confirm p21 expression in juvenile metaphyseal osteoblasts by immunohistochemistry, and no race-, ethnicity- or socially relevant grouping-based analyses were performed.

Population characteristics

The samples were selected based on the availability of juvenile metaphyseal tissue suitable for immunohistochemical analysis. Sex was determined from clinical records. No race, ethnicity or other socially relevant grouping-based analyses were performed because the samples were used only to confirm p21 expression in juvenile metaphyseal osteoblasts by immunohistochemistry.

Recruitment

No participants were recruited specifically for this study. Archival paraffin-embedded human distal femur tissue samples were selected retrospectively based on the availability of juvenile metaphyseal tissue suitable for immunohistochemical analysis. Informed consent was obtained from each participant or their legal guardian, and participants received no compensation.

Ethics oversight

The use of human tissue samples was approved by the Research Ethics Committee of the Graduate School of Medicine and Faculty of Medicine, The University of Tokyo. Informed consent was obtained from each participant or their legal guardian.

Note that full information on the approval of the study protocol must also be provided in the manuscript.

Field-specific reporting

Please select the one below that is the best fit for your research. If you are not sure, read the appropriate sections before making your selection.

☒ Life sciences ☐ Behavioural & social sciences ☐ Ecological, evolutionary & environmental sciences

For a reference copy of the document with all sections, see nature.com/documents/nr-reporting-summary-flat.pdf

Life sciences study design

All studies must disclose on these points even when the disclosure is negative.

Sample size	Biological replicates were at least n=3 unless otherwise noted. The sample size are indicated in the figure legends.<br>No statistical method was used to predetermine sample sizes, but our sample sizes are similar to those reported in previous publications, such as Hoare et al., Nat Cell Biol. 2016; Bird et al., Science translational medicine 2018 and Amor et al., Nature 2020.
Data exclusions	We excluded a few mice which unexpectedly died after tamoxifen treatment. In scRNA-seq analysis, cells with 1,500 < nFeature , 5,000 < nCount < 75,000, and low mitochondrial gene expression (< 20%) were further analyzed.
Replication	All experiments (except for bulk RNA-seq and scRNA-seq) were performed at least 3 times and we were able to confirm the reproducibility of our results.
Randomization	Experimental groups were based on their genotypes.
Blinding	The investigators were not blinded to allocation during experiments or analysis, as group allocation was clearly visible in the sample due to phenotypic changes.

Reporting for specific materials, systems and methods

We require information from authors about some types of materials, experimental systems and methods used in many studies. Here, indicate whether each material, system or method listed is relevant to your study. If you are not sure if a list item applies to your research, read the appropriate section before selecting a response.

Materials & experimental systems

n/a	Involved in the study	
☐	☒	Antibodies
☐	☒	Eukaryotic cell lines
☒	☐	Palaeontology and archaeology
☐	☒	Animals and other organisms
☐	☒	Clinical data
☒	☐	Dual use research of concern
☒	☐	Plants

Methods

n/a	Involved in the study	
☒	☐	ChIP-seq
☐	☒	Flow cytometry
☒	☐	MRI-based neuroimaging

Antibodies

Antibodies used

Primary antibodies for immunostaining and immunofluorescence:
rat anti-EMCN (1:200; SC-65495, Santa Cruz)
rabbit anti-OSX (1:500–1:750; ab209484, Abcam)
rabbit anti-p21 (1:500–1:750; ab188224, Abcam)
rabbit anti-KI67 (1:200; ab16667, Abcam)
rabbit anti-c-MYC (1:200; ab32072, Abcam)
rabbit anti-RUNX2 (1:500–1:750; ab192256, Abcam)
rabbit anti-IHH (1:500–1:1000; ab39634, Abcam)
rabbit anti-phospho-Histone H2A.X (Ser139) (1:500–1:750; 9718, Cell Signaling)
rabbit anti-cleaved caspase-3 (Asp175) (1:200; 9661, Cell Signaling)
rabbit anti-phospho-p53 (Ser15) (1:200–1:500; 9284, Cell Signaling)
rabbit anti-integrin β3 (1:200; 4702, Cell Signaling)
rabbit anti-COL1A1 (1:200; CL50151AP, Cedarlene)
rabbit anti-p-RPA (pS4/S8) (1:400; ab87277, Abcam)

HRP-conjugated secondary antibodies:
Histofine (Nichirei)

Fluorescent secondary antibodies:
Alexa Fluor 647-conjugated donkey anti-rabbit IgG (A31573, Invitrogen)
Alexa Fluor 488-conjugated goat anti-rabbit IgG (A11034, Invitrogen)
Alexa Fluor 647-conjugated goat anti-rat IgG (A21247, Invitrogen)

Antibodies for MACS and fluorescence-activated cell sorting (FACS):
eFluor450-conjugated anti-CD31 (clone 390; Cat. No. 48-0311-82)
eFluor450-conjugated anti-CD45 (clone 30F-11; Cat. No. 48-0451-82)
eFluor450-conjugated anti-TER119 (clone TER119; Cat. No. 48-5921-82)

Validation

The primary antibodies used in this study are commercially available and have been validated by the manufacturers for the indicated

Validation	applications and species. Antibody specificity was further supported by the expected tissue, cellular or subcellular staining patterns observed in this study, and, where applicable, by genetic or reporter-based validation, including p21 reporter overlap.

Eukaryotic cell lines

Policy information about cell lines and Sex and Gender in Research

Cell line source(s)	V6.5 mouse embryonic stem cells (ESCs) were used for the generation of genetically modified mouse lines. V6.5 ESCs were originally derived from F1 hybrid embryos of C57BL/6 and 129/Sv mice and have been described previously.
Authentication	The targeted ESC clones were validated by PCR-based genotyping and sequencing of the targeted genomic loci before blastocyst injection.
Mycoplasma contamination	ESC cultures were confirmed to be negative for mycoplasma contamination.
Commonly misidentified lines (See ICLAC register)	The V6.5 ESC line is not listed in the database of commonly misidentified cell lines maintained by ICLAC.

Animals and other research organisms

Policy information about studies involving animals; ARRIVE guidelines recommended for reporting animal research, and Sex and Gender in Research

Laboratory animals	All mice were housed in a specific pathogen-free animal facility under a 12 h light/12 h dark cycle with a temperature of 20-24 °C and a humidity of 45-65%, with food and water provided ad libitum.<br>Pseudopregnant ICR and male/female ICR (8-10 week old) mice were obtained from Japan SLC to make chimeric mice.<br>C57BL/6 mice were also obtained from Japan SLC.<br>Cdkn1a reporter, Osx reporter, Control, and c-Myc-inducible mice were developed in this laboratory.<br>Rosa LSL-tTA mice were described previously (Taguchi et al., 2025).<br>Col1a1::TetO-cMyc-IRES-mCherry mice were described previously (Hirano et al., 2022).<br>Rosa26-mTmG and Trp53-LSL-R172H mice were obtained from Jackson Laboratories.<br><br>Strain genotype/Source /Genetic background / substrain Age used.<br><br>p21-IRES-CreERT2 knock-in/Generated in this study using V6.5 ESCs/mixed C57BL/6–129×1/Sv/3–8 weeks, E16.5 where indicated<br>Osx-CreERT2 knock-in/Generated in this study using V6.5 ESCs/mixed C57BL/6–129×1/Sv 3–8 weeks, as indicated<br>Rosa26-mTmG reporter/The Jackson Laboratory/mixed after breeding; original strain from JAX/3–8 weeks, E16.5 where indicated<br>Rosa26-LSL-tTA knock-in/Previously described; maintained in-house/mixed C57BL/6–129×1/Sv/3 or 8 weeks for induction experiments<br>Col1a1::TetO-cMyc-IRES-mCherry knock-in/Previously described; maintained in-house/mixed C57BL/6–129×1/Sv/3 or 8 weeks for induction experiments<br>Trp53-LSL-R172H knock-in/The Jackson Laboratory/mixed after breeding; original strain from JAX/mixed C57BL/6–129×1/Sv 3–8 weeks, as indicated<br>p21-IRES-CreERT2; Rosa26-mTmG; Atm KO chimeric mice/Generated in this study by CRISPR/Cas9-mediated editing of ESCs followed by blastocyst injection/mixed/chimeric background age indicated in figures<br>C57BL/6J wild-type mice/Japan SLC C57BL/6J/3, 6 or 8 weeks, as indicated<br>ICR mice/Japan SLC/ICR/8-week-old females for blastocyst preparation/transfer
Wild animals	N/A
Reporting on sex	We used both male and female mice.<br>For transcriptomic analysis (RNA-seq and scRNA-seq), only males were used.
Field-collected samples	N/A
Ethics oversight	All animal experiments were approved by the Animal Experiment Committees of the Institute of Medical Science (IMSUT) and the Graduate School of Medicine, The University of Tokyo. All animal care and procedures were conducted in accordance with institutional guidelines.

Note that full information on the approval of the study protocol must also be provided in the manuscript.

Clinical data

Policy information about clinical studies

All manuscripts should comply with the ICMJE guidelines for publication of clinical research and a completed CONSORT checklist must be included with all submissions.

Clinical trial registration	Not applicable. This study did not involve a clinical trial; human tissue samples were used only for immunohistochemical analysis.
Study protocol	N/A

Data collection | N/A

Outcomes | N/A

Plants

Seed stocks: N/A

Novel plant genotypes: N/A

Authentication: N/A

Flow Cytometry

Plots

Confirm that:

☒ The axis labels state the marker and fluorochrome used (e.g. CD4-FITC).

☒ The axis scales are clearly visible. Include numbers along axes only for bottom left plot of group (a 'group' is an analysis of identical markers).

☒ All plots are contour plots with outliers or pseudocolor plots.

☒ A numerical value for number of cells or percentage (with statistics) is provided.

Methodology

Sample preparation

mGFP+ osteoblasts were isolated from 3- and 8-week-old Osx-CreERT2; mTmG mice for RNA-seq analysis. Two femurs and two tibias were dissected from each mouse, and surrounding soft tissues were carefully removed. For control samples, femurs and tibias from two male mice were pooled per replicate. For c-Myc-induced and c-Myc + Trp53-R172H osteoblasts, mGFP+ cells were isolated from Osx-CreERT2; mTmG; LSL-tTA; Col1a1::TetO–c-Myc and Osx-CreERT2; mTmG; LSL-tTA; Col1a1::TetO–c-Myc; Trp53-LSL-R172H mice, respectively (both male and female), 7 days after tamoxifen administration.
To isolate cells specifically from the metaphyseal region, the epiphysis was carefully removed at the midpoint of the growth plate using forceps. Residual chondrocytes from the growth plate were thoroughly removed under a stereomicroscope. The diaphysis was then trimmed approximately 2 mm (for 3- and 8-week-old Osx-CreERT2; mTmG mice) or 5 mm (for 5-week-old Osx-CreERT2; mTmG; LSL-tTA; Col1a1::TetO–c-Myc and Osx-CreERT2; mTmG; LSL-tTA; Col1a1::TetO–c-Myc; Trp53-LSL-R172H mice) distal to the growth plate.
The harvested metaphyseal regions from distal femurs and proximal tibias were transferred to digestion buffer comprising M199 with Hanks' salts (Gibco) containing 250 U/mL type I collagenase (Worthington), 250 U/mL type II collagenase (Worthington), 100 U/mL DNase I (1 mg/mL; Roche), 1 mg/mL poloxamer 188 (Sigma), 1 mg/mL BSA (Fujifilm), and 20 mM HEPES (Nacalai Tesque). The bones were placed in a mortar with 1 mL of digestion buffer and crushed using a pestle for at least 1 minute. The supernatant was collected in a 15 mL conical tube. This process was repeated until the bones appeared completely white (typically three times or more).
The remaining bone fragments were pooled into the same tube and supplemented with digestion buffer to a final volume of 5mL per sample. Samples were incubated at 37°C for 30 minutes at 210rpm. Following digestion, the supernatant was filtered through a 70µm cell strainer into a 50mL tube containing 5–10mL of ice-cold cell suspension buffer [Ca2+/Mg2+-free PBS (Nacalai Tesque) containing 2% fetal bovine serum, 1mg/mL poloxamer 188, and 100U/mL penicillin/streptomycin (Gibco)].
Bone fragments were further digested with 3–5mL of prewarmed digestion buffer, and the crushing–filtration process was repeated 4–5 times. Red blood cells were lysed using ACK lysis buffer (Gibco). Bone chips were removed, and single-cell suspensions were obtained by filtering through a 40µm strainer for subsequent magnetic-activated cell sorting (MACS) preparation.

Instrument: FACS Melody (BD Biosciences) were used to collect data.

Software: FACSChorus v3.0 were used to collect data. FlowJo v10.10.0 was used for analyzing the cell frequencies.

Cell population abundance: Around 15-25% of the single cells were mGFP+ cells.

Gating strategy

Single cells were gated by using FSC-A vs SSC-A, SSC-H vs SSC-W and FSC-H vs FSC-W parameters. mGFP+ were sorted for negative gates by eFluor450-labeled CD31, CD45, and TER119 after dead cell removal by 7AAD. For mGFP+ populations were identified by comparing to the control samples from control mice.

☒ Tick this box to confirm that a figure exemplifying the gating strategy is provided in the Supplementary Information.
