# Supplementary material for: Inherent tissue homeostasis of the juvenile metaphysis provides a foundation for osteosarcoma development
Source: Nat Commun. 2026 Jun 26;17:6241. doi: 10.1038/s41467-026-74929-6 (PMC13373234; doi:10.1038/s41467-026-74929-6)
Supplement: Supplementary file 3 — Transparent Peer Review file [file 41467_2026_74929_MOESM3_ESM.pdf]

nature portfolio

Peer Review File

Inherent tissue homeostasis of the juvenile metaphysis provides a foundation for osteosarcoma development

Corresponding Author: Professor Yasuhiro Yamada

This file contains all reviewer reports in order by version, followed by all author rebuttals in order by version.

Version 0:

Reviewer comments:

Reviewer #1

(Remarks to the Author)
The revised manuscript has been substantially strengthened, accompanied by additional experimental data. Notably, the authors added several key experiments using Cdkn1a-CreERT2 to support the conclusion, including scRNA-seq, lineage tracing, and tumor induction with cMyc plus p53 mutations. Listed below are my additional thoughts on the authors' responses.

1. RE: scRNA-seq of Cdkn1a-CreERT2+ cells: The findings confirm the heterogeneity of p21+ cells in the juvenile metaphysis, encompassing a broad spectrum of osteoblasts, osteoblast precursors/pre-osteoblasts, and BMSCs. The authors should include the flow cytometry plot and gating strategy of the mTmG reporter-positive cells in the Extended Data, which is known to be technically challenging. The authors should also include the "parental" scRNA-seq data before subsetting, which should also include at least some contaminating hematopoietic cells. The percentage of mesenchymal cells (osteoblasts and BMSCs) among the entire p21-mT/mG+ cells in the bone marrow will be an important piece of information to accurately interpret the current data.

2. RE: Evidence of replication stress in p21-mGFP+ cells: The authors did not respond appropriately to demonstrate whether doxorubicin treatment increases metaphyseal mGFP+ cells. Given that c-Myc expression can be triggered by numerous signals, including growth factors, it is essential to demonstrate that metaphyseal p21+ cells can be induced by DNA damage. This would be considered a key piece of evidence.

3. RE: Link between p21 and Hedgehog: Without a genetic proof, I feel that the study overstates the importance of IHH in c-Myc driven proliferation, particularly at the beginning of the Discussion. It is still possible that other non-growth plate Hedgehog sources play a primary role in inducing p21+ metaphyseal cells. The authors should appropriately adjust the claim to reflect the findings.

4. RE: Tumor induction by p21+ cells: The authors provided critical evidence that osteosarcoma-like lesions can be induced by p21+ cells. However, the entirety of these important data is currently relegated to Extended Data Figure 11. Some of the most representative images should be transposed to the main Figure 5 to increase visibility.

- Line 104: ...in a small subset of growth plate chondrocytes [in the prehypertrophic zone].
- Line 106, mGFP+ cells "frequently" expressed OSX: The authors should include the percentage of OSX+ cells among mGFP+ cells.
- Line 112, ...displayed "frequent" expression of p21 protein: The number should be included.
- Line 137, these cells give rise "predominantly" to differentiated cells: Again, the number should be provided to support this statement.
- Line 141, p21+ cells "often" expressed gH2AX: The authors should quantify the percentage of gH2AX+ cells among p21+ cells. The number should be explicitly stated in the main text.
- Line 226: Fig.4F, instead of Fig.4H
- Line 227: Fig.4G, instead of Fig.4I

Reviewer #2

(Remarks to the Author)

Reviewer #3

(Remarks to the Author)
The manuscript by Saito et al. provides experimental evidence supporting the hypothesis that deregulated proliferation of juvenile metaphyseal cells contributes to the development of osteosarcoma. The authors propose that intrinsic replication stress responses in juvenile osteoblasts serve as a protective mechanism against malignant transformation. Finally, they suggest that Myc amplification and p53 inactivation facilitate osteosarcoma initiation.

While the results presented are of potential value, several conclusions are overstated and not fully supported by the data. In particular, measurements of p21 and γH2AX levels do not constitute direct evidence of replication stress. The authors report correlations between elevated Ki67/mGFP+ cell ratios, increased p21 and γH2AX levels in juvenile metaphyseal tissue (Figs. 1 and 3), reductions of these markers in the absence of ATM (Fig. 2), and enhancement upon Myc overexpression (Fig. 5). However, these observations primarily reflect activation of the DNA damage response (DDR) rather than replication stress per se.

To convincingly demonstrate replication stress in proliferating osteoblasts, additional experiments should directly measure replication dynamics. For instance, EdU or BrdU incorporation assays could be coupled with established replication stress readouts such as phosphorylated RPA or ssDNA detection. The spatial distribution of γH2AX foci in S-phase cells would also be informative. Although the authors attempted to assess EdU and γH2AX signals (Fig. 2D), the presented images lack sufficient resolution and magnification to judge foci formation reliably, and quantification is absent. Moreover, γH2AX signals often appear in EdU-negative cells, and in mGFP(p21)-positive cells, the γH2AX signal appears pan-nuclear—suggestive of apoptosis rather than replication stress.

Classical replication stress first activates the ATR signaling pathway (see Nat Cell Biol. 2014;16:2–9). When replication stress overwhelms this response, ATM and other kinases are subsequently engaged; ATM activation during S phase can stabilize p53 and promote apoptosis (PMIDs: 10801407, 11331603). High p21 levels may therefore reflect a path to cell death during normal tissue differentiation and remodeling, consistent with pan-nuclear γH2AX and phosphorylated p53 signals. The current findings support this interpretation, particularly under ATM deficiency conditions.

Specific comments:
• Evidence for replication stress is insufficient. The data mainly demonstrate DDR activation in the metaphysis at 3–4 weeks, correlating with increased EdU incorporation, but causality remains unproven. All references to γH2AX and p21 as “replication stress markers” should be revised; with the available data, they serve only as DDR indicators.
• In Extended Data Fig. 3G, EdU and OSX are shown, but not γH2AX; this should be corrected in the text.
• The statement “Replication stress induces p21 in immature osteoblasts” would be more accurately phrased as “Findings are consistent with replication stress–induced p21 expression,” unless direct replication stress assays (e.g., DNA fiber assays, RPA foci quantification) are included.
• The role of p53 should be clarified: it primarily functions to couple replication stress to cell-cycle arrest or apoptosis, not to prevent stress initiation. Cancer cells often tolerate a “chronic stress without checkpoint” state.

Major concern:
In Fig. 1D, the mGFP-p21 reporter signal appears mainly cytoplasmic, whereas endogenous p21 by immunofluorescence is predominantly nuclear. This discrepancy should be explicitly discussed, with separate fluorescence channels shown for clarity in key figures.

Overall, Saito et al. provide useful observations linking osteoblast proliferation, DDR activation, and osteosarcoma formation. During metaphyseal development, osteoblasts undergo proliferation, differentiation, and programmed cell death—processes tightly regulated by p53 and its effectors. DNA replication errors are common during rapid proliferation, and if DNA damage exceeds repair capacity, cell death ensures tissue homeostasis. Disruption of the balance between proliferation, differentiation, and cell death can lead to genome instability and transformation. The data from Saito et al. reinforce this model, illustrating that Myc overexpression and p53 loss increase replication-associated stress and DDR activation, ultimately promoting osteosarcoma formation. However, these findings represent an extension rather than a conceptual advance, as similar mechanisms have been well documented in other tissues and models. By this, I do not diminish the potential value and interest that some NCOMM readers may appreciate in the results presented by Saito et al.

Version 1:

Reviewer comments:

Reviewer #1

(Remarks to the Author)
The authors have responded to my queries in a satisfactory manner. Congratulations on this beautiful and comprehensive study.

Reviewer #3

(Remarks to the Author)
The authors have satisfactorily addressed all my concerns from the previous revision. I agree that the manuscript has improved in the current version. I have no further comments.

Open Access This Peer Review File is licensed under a Creative Commons Attribution 4.0 International License, which permits use, sharing, adaptation, distribution and reproduction in any medium or format, as long as you give appropriate credit to the original author(s) and the source, provide a link to the Creative Commons license, and indicate if changes were made.
In cases where reviewers are anonymous, credit should be given to 'Anonymous Referee' and the source.
The images or other third party material in this Peer Review File are included in the article's Creative Commons license, unless indicated otherwise in a credit line to the material. If material is not included in the article's Creative Commons license and your intended use is not permitted by statutory regulation or exceeds the permitted use, you will need to obtain permission directly from the copyright holder.
To view a copy of this license, visit https://creativecommons.org/licenses/by/4.0/

Response to Referees' comments:

Reviewer #1 (Remarks to the Author):

We sincerely thank the reviewer for the careful re-evaluation of our revised manuscript and for the constructive and thoughtful comments provided throughout the review process.

Comment:
The revised manuscript has been substantially strengthened, accompanied by additional experimental data. Notably, the authors added several key experiments using Cdkn1a-CreERT2 to support the conclusion, including scRNA-seq, lineage tracing, and tumor induction with cMyc plus p53 mutations. Listed below are my additional thoughts on the authors' responses.

Response:
We thank the reviewer for the careful evaluation of our revised manuscript and for the positive assessment of the additional experiments included in the revision. We particularly appreciate the reviewer's recognition of the newly added analyses using the *Cdkn1a-CreERT2* system, including scRNA-seq, lineage tracing, and tumor induction experiments with *c-Myc* expression and p53 inactivation. We are encouraged that the reviewer found these experiments to substantially strengthen the study. We also thank the reviewer for the additional constructive comments and suggestions, which have helped us further improve the manuscript.

Comment:

1. *RE: scRNA-seq of Cdkn1a-CreERT2+ cells: The findings confirm the heterogeneity of p21+ cells in the juvenile metaphysis, encompassing a broad spectrum of osteoblasts, osteoblast precursors/pre-osteoblasts, and BMSCs. The authors should include the flow cytometry plot and gating strategy of the mTmG reporter-positive cells in the Extended Data, which is known to be technically challenging. The authors should also include the "parental" scRNA-seq data before subsetting, which should also include at least some contaminating hematopoietic cells. The percentage of mesenchymal cells (osteoblasts and BMSCs) among the entire p21-mT/mG+ cells in the bone marrow will be an important piece of information to accurately interpret the current data.*

Response:
We thank the reviewer for this helpful suggestion. In response, we have now included the strategy for isolation of *mTmG* reporter-positive cells, which includes a MACS procedure, flow cytometry plots, and detailed gating strategy in the revised **Extended Data Fig. 3F**. In addition, we have now included the "parental" scRNA-seq dataset prior to mesenchymal cell subsetting in **Extended Data Fig. 3G**. As the reviewer anticipated, this dataset contained a subset of contaminating hematopoietic cells, particularly erythroid-lineage cells. These contaminating populations were identified based on canonical hematopoietic markers and excluded from downstream analyses. Regarding the percentage of mesenchymal cells among the entire *p21-mT/mG*+ cell population in the bone marrow, we note that precise quantification is technically difficult in the current experimental setting because CD45- and Ter119-positive cells were depleted by MACS, followed by FACS depletion of CD45-, CD31-, and Ter119-positive cells, prior to scRNA-seq analysis. Therefore, the analyzed cell population was already enriched for non-hematopoietic mesenchymal cells before sequencing.

Comment:

2. *RE: Evidence of replication stress in p21-mGFP+ cells: The authors did not respond appropriately to demonstrate whether doxorubicin treatment increases metaphyseal mGFP+ cells. Given that c-Myc expression can be triggered by numerous signals, including growth factors, it is essential to demonstrate that metaphyseal p21+ cells can be induced by DNA damage. This would be considered a key piece of evidence.*

Response:
We thank the reviewer for this important suggestion. In response, we performed additional experiments to directly examine whether DNA damage induces metaphyseal p21+ cells. We found that doxorubicin treatment significantly increased the number of p21+ cells in the juvenile metaphysis, supporting the idea that these cells can indeed be induced by DNA damage. In contrast, we did not observe a comparable increase in p21+ cells in adult mice following doxorubicin treatment. These findings further emphasize the distinct properties of juvenile versus adult osteoblasts and support the notion that juvenile metaphyseal osteoblasts exhibit a unique stress-responsive state associated with rapid bone growth and proliferation. These new data have been added to **Extended Data Fig. 4F**.

In addition, to further distinguish replication stress-associated DDR activation from a generally heightened sensitivity to DNA damage, we analyzed replication stress-associated markers in proliferating metaphyseal cells. Replication stress can arise from diverse DNA replication-intermediate, including single-ended DSBs generated when replication forks encounter single-strand breaks, ssDNA gaps and DSBs formed following replication fork stalling or collapse, and reversed fork structures such as "chicken foot" intermediates. A common feature of these replication-associated lesions is the accumulation and phosphorylation of RPA on ssDNA regions, including ssDNA gaps and resected DSB ends. Therefore, we used phosphorylated RPA as a marker of replication stress. Because RPA phosphorylation can also occur in contexts other than DNA replication, we specifically assessed p-RPA in cells identified as being in S phase by EdU incorporation. Using this approach, we found that p-RPA- and γH2AX-positive cells were highly enriched among EdU-positive S-phase cells in the juvenile metaphysis. Notably, both γH2AX and p-RPA signals exhibited punctate nuclear staining patterns rather than diffuse pan-nuclear staining, supporting the notion that DNA damage arises during DNA replication in S phase. Together, these findings support the interpretation that actively proliferating juvenile osteoblasts undergo DDR activation during S phase, rather than simply exhibiting nonspecific susceptibility to DNA damage. Importantly, p-RPA expression was reduced in adult metaphyseal cells but further enhanced in *c-Myc*-induced osteoblasts, further strengthening our conclusion. These new data have been added to the revised **Figures 2D**, **3D**, and **5F**, as well as **Extended Data Figs. 4E** and **9D**.

Comment:

3. *RE: Link between p21 and Hedgehog: Without a genetic proof, I feel that the study overstates the importance of IHH in c-Myc driven proliferation, particularly at the beginning of the Discussion. It is still possible that other non-growth plate Hedgehog sources play a primary role in inducing p21+ metaphyseal cells. The authors should appropriately adjust the claim to reflect the findings.*

Response:

We thank the reviewer for this thoughtful comment and agree that genetic evidence would provide a stronger basis for establishing a direct causal link between IHH signaling and induction of p21+ metaphyseal cells. In the current study, although our data support an association between Hedgehog signaling and proliferation of juvenile metaphyseal osteoblasts, we acknowledge that we have not formally demonstrated this relationship genetically. We also agree that non–growth plate sources of Hedgehog ligands may potentially contribute to the observed phenotype. In response to the reviewer's suggestion, we have revised and toned down the corresponding statements throughout the manuscript to more carefully reflect the scope and limitations of the current findings. The revised text now emphasizes that our data are consistent with a role for Hedgehog signaling in this process, while acknowledging that the precise cellular source and causal contribution remain to be established in future studies. (**Lines 357-366**)

Comment:

4. *RE: Tumor induction by p21+ cells: The authors provided critical evidence that osteosarcoma-like lesions can be induced by p21+ cells. However, the entirety of these important data is currently relegated to Extended Data Figure 11. Some of the most representative images should be transposed to the main Figure 5 to increase visibility.*

Response:

We thank the reviewer for this valuable suggestion and for recognizing the importance of the tumor induction experiments using the *Cdkn1a-CreERT2* system. We agree that these data represent a key finding of the study and deserve greater visibility in the main presentation of the manuscript. In response, we have moved representative images from the Extended Data Figure to the main **Figure 5I** and **6C** in the revised manuscript. We believe that this reorganization improves the clarity and impact of the study and allows readers to more readily appreciate the significance of the tumor induction experiments.

Comment:

- Line 104: ...in a small subset of growth plate chondrocytes [in the prehypertrophic zone].

Response:

We thank the reviewer for this helpful suggestion. We have revised the text as suggested to clarify that the signal was observed in a small subset of growth plate chondrocytes in the prehypertrophic zone (**Line 107**).

Comment:

- Line 106, mGFP+ cells "frequently" expressed OSX: The authors should include the percentage of OSX+ cells among mGFP+ cells.

Response:
We thank the reviewer for this suggestion. In response, we have now included the percentage of OSX+ cells among mGFP+ cells in the main text (47.1 ± 12.4%; **Lines 109-110**). In addition, we added a new subsection entitled "Quantification of immunofluorescence staining" to the Methods section, describing the procedures used for quantification of immunofluorescence-positive cells.

Comment:
- Line 112, ...displayed "frequent" expression of p21 protein: The number should be included.

Response:
We thank the reviewer for this suggestion. In response, we have now included the percentage of p21+ cells among mGFP+ cells in the main text (34.2±6.9 %; **Line 115**).

Comment:
- Line 137, these cells give rise "predominantly" to differentiated cells: Again, the number should be provided to support this statement.

Response:
We thank the reviewer for this suggestion. In response, we have now included the percentage of mGFP+ cells among FABP4+ cells in the main text (58.7±5.6 %; **Lines 143-144**).

Comment:
- Line 141, p21+ cells "often" expressed γH2AX: The authors should quantify the percentage of γH2AX+ cells among p21+ cells. The number should be explicitly stated in the main text.

Response:
We thank the reviewer for this suggestion. In response, we have now included the percentage of γH2AX+ cells among *p21*-mGFP+ cells in the main text (11.2±5.6 %; **Lines 147-148**). Given that only a subset of *p21*-mGFP+ cells were positive for γH2AX, we also revised the wording from "often" to "a subset of."

Comment:
- Line 226: Fig.4F, instead of Fig.4H
- Line 227: Fig.4G, instead of Fig.4I

Response:
We thank the reviewer for pointing out these errors (**Line 234** and **Line 235**). We have corrected the figure references accordingly.

Finally, we sincerely thank the reviewer for the constructive comments and thoughtful suggestions throughout the review process. The reviewer's feedback substantially improved the clarity, rigor, and presentation of the manuscript. We greatly appreciate the reviewer's careful evaluation and supportive comments regarding the revised study.

Reviewer #3 (Remarks to the Author):

We sincerely thank the reviewer for the careful evaluation of our manuscript and for the insightful and constructive comments. We particularly appreciate the reviewer's thoughtful suggestions regarding the interpretation of replication stress and DDR activation, which helped us substantially strengthen both the experimental rigor and conceptual clarity of the study.

Comment:
The manuscript by Saito et al. provides experimental evidence supporting the hypothesis that deregulated proliferation of juvenile metaphyseal cells contributes to the development of osteosarcoma. The authors propose that intrinsic replication stress responses in juvenile osteoblasts serve as a protective mechanism against malignant transformation. Finally, they suggest that Myc amplification and p53 inactivation facilitate osteosarcoma initiation.

While the results presented are of potential value, several conclusions are overstated and not fully supported by the data. In particular, measurements of p21 and γH2AX levels do not constitute direct evidence of replication stress. The authors report correlations between elevated Ki67/mGFP+ cell ratios, increased p21 and γH2AX levels in juvenile metaphyseal tissue (Figs. 1 and 3), reductions of these markers in the absence of ATM (Fig. 2), and enhancement upon Myc overexpression (Fig. 5). However, these observations primarily reflect activation of the DNA damage response (DDR) rather than replication stress per se.

To convincingly demonstrate replication stress in proliferating osteoblasts, additional experiments should directly measure replication dynamics. For instance, EdU or BrdU incorporation assays could be coupled with established replication stress readouts such as phosphorylated RPA or ssDNA detection. The spatial distribution of γH2AX foci in S-phase cells would also be informative. Although the authors attempted to assess EdU and γH2AX signals (Fig. 2D), the presented images lack sufficient resolution and magnification to judge foci formation reliably, and quantification is absent. Moreover, γH2AX signals often appear in EdU-negative cells, and in mGFP(p21)-positive cells, the γH2AX signal appears pan-nuclear—suggestive of apoptosis rather than replication stress.

Classical replication stress first activates the ATR signaling pathway (see Nat Cell Biol. 2014;16:2–9). When replication stress overwhelms this response, ATM and other kinases are subsequently engaged; ATM activation during S phase can stabilize p53 and promote apoptosis (PMIDs: 10801407, 11331603). High p21 levels may therefore reflect a path to cell death during normal tissue differentiation and remodeling, consistent with pan-nuclear γH2AX and phosphorylated p53 signals. The current findings support this interpretation, particularly under ATM deficiency conditions.

Response:
We thank the reviewer for these highly insightful and important comments regarding the interpretation of replication stress in our study. We agree that p21 and γH2AX alone do not constitute definitive evidence of replication stress and appreciate the reviewer's careful distinction between replication stress and general DDR activation. The reviewer's comments have helped us substantially strengthen both the experimental rigor and the interpretation of our findings.

In response, we performed additional experiments to more directly assess replication stress in juvenile metaphyseal osteoblasts. Specifically, we conducted immunostaining for phosphorylated RPA, a marker associated with replication stress as suggested, in conjunction with EdU incorporation to identify S-phase cells. As described below, these additional analyses further support the presence of replication stress in proliferating juvenile osteoblasts and strengthen the central conclusions of the manuscript.

Specific comments:
Comment:
• Evidence for replication stress is insufficient. The data mainly demonstrate DDR activation in the metaphysis at 3–4 weeks, correlating with increased EdU incorporation, but causality remains unproven. All references to γH2AX and p21 as "replication stress markers" should be revised; with the available data, they serve only as DDR indicators.

Response:
We thank the reviewer for this important comment regarding the interpretation of replication stress versus general DDR activation. In response, we performed additional analyses using phosphorylated RPA (p-RPA S4/S8) staining and quantified p-RPA-positive nuclei in juvenile metaphyseal cells (**Figure 2D**).

These analyses revealed that p-RPA-positive cells were highly enriched among EdU-positive S-phase cells, indicating that DNA damage arises during S phase in association with active proliferation of juvenile osteoblasts. Furthermore, the number of p-RPA-positive cells markedly decreased following cessation of bone growth, consistent with the age-dependent reduction in proliferative activity (**Figure 3D** and **Extended Data Fig. 4E**). In addition, *c-Myc* induction significantly increased the frequency of p-RPA-positive cells in the metaphysis, consistent with the established role of *c-Myc* in enhancing replication stress (**Figure 5F** and **Extended Data Fig. 9D**).

We also added higher-resolution images of EdU-labeled S-phase cells stained for γH2AX, together with quantification of their frequency (**Figure 2D**). We respectfully ask the reviewer to note that these analyses were performed on mouse tissue sections rather than *in vitro* cultured cells, and therefore, further improvement in image resolution is technically limited. We also note that, because the samples are tissue sections, it is not feasible to integrate the full z-axis information of each nucleus by image projection. Thus, while EdU signals in some sections may appear to delineate the nuclear contour, this does not necessarily indicate labeling of the entire nuclear volume. Nevertheless, the newly added images demonstrate that γH2AX signals in EdU-positive cells predominantly exhibit punctate nuclear foci rather than pan-nuclear staining, supporting their association with replication-associated DNA damage responses rather than apoptotic nuclear staining.

Together with our findings that EdU-positive S-phase cells frequently express p-RPA and γH2AX, these findings support the interpretation that the observed p21 and γH2AX expression patterns are not merely reflecting nonspecific DDR activation, but are associated with replication stress occurring in proliferating juvenile osteoblasts. We have revised the manuscript accordingly and now describe p21 and γH2AX more carefully within the context of replication stress-associated DDR responses.

Comment:
• *In Extended Data Fig. 3G, EdU and OSX are shown, but not γH2AX; this should be corrected in the text.*

Response:
We thank the reviewer for pointing out this issue. In the revised manuscript, we have added additional analyses demonstrating co-localization of EdU-positive cells with γH2AX and p-RPA expression, together with quantification of their frequencies (**Figure 2D**).

Together with our previous observation that most EdU-positive proliferating cells in the juvenile metaphysis expressed the osteoblast marker OSX, these findings support the interpretation that proliferating juvenile osteoblast-lineage cells undergo DNA replication-associated DDR activation during metaphyseal growth. These data are presented in the revised **Figure 2D** and **Extended Data Fig. 3I**.

Comment:
• *The statement "Replication stress induces p21 in immature osteoblasts" would be more accurately phrased as "Findings are consistent with replication stress–induced p21 expression," unless direct replication stress assays (e.g., DNA fiber assays, RPA foci quantification) are included.*

Response:
We thank the reviewer for this important suggestion. In response, we performed additional analyses including quantification of p-RPA-positive cells, which were consistent with the presence of replication stress in proliferating juvenile osteoblasts. However, we agree that the original wording was overly definitive given the nature of the available evidence.

Accordingly, we have revised and toned down the relevant statements throughout the manuscript to more carefully describe our findings as being consistent with replication stress-associated p21 expression and DDR activation, rather than making a definitive causal claim.

Comment:
• *The role of p53 should be clarified: it primarily functions to couple replication stress to cell-cycle arrest or apoptosis, not to prevent stress initiation. Cancer cells often tolerate a "chronic stress without checkpoint" state.*

Response:
We thank the reviewer for this important conceptual clarification regarding the role of p53 in replication stress responses. We think that p53 primarily functions downstream of replication stress and DDR signaling to induce

cell-cycle arrest, apoptosis, or other checkpoint responses, rather than preventing stress initiation itself. In response to the reviewer's comment, we have revised the manuscript to more clearly distinguish replication stress from the p53-dependent cellular responses that follow. The revised text now emphasizes that p53-mediated checkpoint activation likely functions as a protective mechanism that limits the propagation of stressed proliferating osteoblasts, whereas loss of p53 permits continued proliferation under DNA replication-associated stressed conditions, thereby promoting osteosarcoma development. (**Lines 381-387**)

Comment:
Major concern:
In Fig. 1D, the mGFP-p21 reporter signal appears mainly cytoplasmic, whereas endogenous p21 by immunofluorescence is predominantly nuclear. This discrepancy should be explicitly discussed, with separate fluorescence channels shown for clarity in key figures.

Response:
We thank the reviewer for raising this point. We would like to clarify that the mGFP signal in the *p21* reporter system does not represent the subcellular localization of endogenous p21 protein. In this reporter model, mGFP is membrane-targeted and is therefore expected to localize predominantly to the cell membrane/cytoplasm compartment, whereas endogenous p21 protein is detected mainly in the nucleus by immunofluorescence. Thus, the apparent difference in localization between mGFP and endogenous p21 protein does not reflect a discrepancy between the reporter and endogenous p21 expression, but rather the distinct design and localization properties of the membrane-targeted reporter protein. We have now added this explanation to the revised manuscript. (**Lines 94–95**)

Comment:
Overall, Saito et al. provide useful observations linking osteoblast proliferation, DDR activation, and osteosarcoma formation. During metaphyseal development, osteoblasts undergo proliferation, differentiation, and programmed cell death—processes tightly regulated by p53 and its effectors. DNA replication errors are common during rapid proliferation, and if DNA damage exceeds repair capacity, cell death ensures tissue homeostasis. Disruption of the balance between proliferation, differentiation, and cell death can lead to genome instability and transformation. The data from Saito et al. reinforce this model, illustrating that Myc overexpression and p53 loss increase replication-associated stress and DDR activation, ultimately promoting osteosarcoma formation. However, these findings represent an extension rather than a conceptual advance, as similar mechanisms have been well documented in other tissues and models. By this, I do not diminish the potential value and interest that some NCOMM readers may appreciate in the results presented by Saito et al.

Response:
We appreciate the reviewer's thoughtful perspective regarding the conceptual positioning of our study. As pointed out by the reviewer, previous studies in other tissues and experimental systems have established important links between deregulated proliferation, replication-associated stress, DDR activation, and tumorigenesis. We agree that our original manuscript did not sufficiently distinguish replication stress from general DDR activation.

Importantly, however, based on the reviewer's constructive suggestions, we have now incorporated additional experiments, including p-RPA staining and higher-resolution analyses of γH2AX staining patterns in S-phase cells, which further support the presence of replication stress in juvenile osteoblasts and strengthen the central conclusions of the study (**Figure 2D**, **3D**, **5F**, **Extended Data Fig. 4E**, **9D**).

In this revised framework, we would like to emphasize that our study suggests that replication stress-associated responses arise physiologically in normal juvenile osteoblasts during metaphyseal growth and may function as an intrinsic tumor-suppressive mechanism through activation of p53. Furthermore, our data suggest that disruption of this protective mechanism by p53 deficiency promotes osteosarcoma initiation. We believe that the identification of replication stress-associated tumor suppression in normal juvenile osteoblasts represents an important conceptual aspect of the study and may provide insight into the age and anatomical specificity of osteosarcoma development.

Finally, we sincerely thank the reviewer for the highly insightful and constructive comments. The reviewer's careful evaluation substantially improved both the experimental rigor and conceptual clarity of the manuscript, particularly regarding the interpretation of replication stress and DDR activation in juvenile osteoblasts. We believe that the additional experiments, revised analyses, and toned-down interpretations have significantly strengthened the study, and we greatly appreciate the reviewer's thoughtful input and expertise.
